# Supplementary material for: Variation in local trust Do Not Attempt Cardiopulmonary Resuscitation (DNACPR) policies: a review of 48 English healthcare trusts
Source: BMJ Open. 2015 Jan 13;5(1):e006517. doi: 10.1136/bmjopen-2014-006517 (PMC4298091; doi:10.1136/bmjopen-2014-006517)
Supplement: Author's manuscript [file bmjopen-2014-006517.draft_revisions.pdf]

BMJ Open

# Variation in local Trust Do Not Attempt Cardiopulmonary Resuscitation policies: a review of 48 English health care trusts



| | |
|---|---|
| Journal: | *BMJ Open* |
| Manuscript ID: | bmjopen-2014-006517 |
| Article Type: | Research |
| Date Submitted by the Author: | 01-Sep-2014 |
| Complete List of Authors: | Freeman, Karoline; Warwick Medical School,<br>Field, Richard; Heartlands Hospital,<br>Perkins, Gavin; University of Warwick, Clinical Trials Unit |
| <b>Primary Subject Heading</b>: | Health policy |
| Secondary Subject Heading: | Emergency medicine, Communication, Health policy, Intensive care, Palliative care |
| Keywords: | INTERNAL MEDICINE, MEDICAL ETHICS, PALLIATIVE CARE, Adult intensive & critical care < INTENSIVE & CRITICAL CARE, Health policy < HEALTH SERVICES ADMINISTRATION & MANAGEMENT, Clinical governance < HEALTH SERVICES ADMINISTRATION & MANAGEMENT |
| | |



SCHOLARONE™ Manuscripts

For peer review only

# Variation in local Trust Do Not Attempt Cardiopulmonary Resuscitation policies: a review of 48 English health care trusts

Karoline Freeman[1], Richard A. Field[1,2], Gavin D. Perkins[1,2]

[1] Warwick Medical School, University of Warwick, Coventry, CV4 7AL

[2] Heart of England NHS Foundation Trust, Bordesley Green East, Birmingham, B9 5SS

Corresponding author:

## Abstract

**Background:** National guidelines for do not attempt cardiopulmonary resuscitation (DNACPR) decisions exist, but implementation is dependent on local policy. This study examined DNACPR policies from English acute, community and ambulance service Trusts to explore consistencies and variation in implementation of national guidelines between healthcare organisations.

**Methods:** Freedom of information requests for adult DNACPR policies were sent to a random sample of 48 English health care Trusts. DNACPR policies were assessed on aspects identified from national guidelines including documentation, ethical and legal issues, decision makers and involvement of others in DNACPR decisions as well as practical considerations such as validity, review and portability of decisions.

**Results:** Policies from 26 acute, 12 community and 10 ambulance service Trusts were reviewed. There was variation in terminology used (85% described documents as policies, 6% procedures and 8% guidelines). Only one quarter of Trusts used the recommended Resuscitation Council (UK) record form (or a modification of the form). There was variation in the terminology used which included DNAR, DNACPR, Not for CPR and AND (allow natural death). Accountability for DNACPR decisions rested with consultants at all acute Trusts and the most senior clinician at community Trusts. Most trusts (74%) recommended discussion of decisions with a multi-disciplinary team. Compliance with guidance requiring clinical staff to assess the



For peer review only - http://bmjopen.bmj.com/site/about/guidelines.xhtml

patient for capacity and when to consult a lasting power of attorney or independent mental capacity advocate occurred less commonly. There was wide variation in the duration of time over which a DNACPR decision was considered valid as well as in the Trusts' approach to reviewing DNACPR decisions. The level of portability of DNACPR decisions between health care organisations was one of the greatest sources of variation.

**Conclusions:** There is significant variation in the translation of the national DNACPR guidelines into English health care Trusts DNACPR policies.

**Keywords: Do not attempt cardiopulmonary resuscitation, DNACPR, policy, national guideline, review**

**Strengths and limitations of this study**

- The study evaluated a representative sample of DNACPR policies and procedures from Acute, Community and Ambulance Service Trusts in England
- The study identified marked variation in NHS organisations approaches to DNACPR policy and practice
- Key areas for strengthening current approaches were identified and included improving consistency for recording decisions and ensuring decisions are transferable between healthcare settings
- Whether improving consistency in DNACPR policies will translate to improved implementation into practice and patient experience requires further study
- The findings require confirmation in other settings



For peer review only - http://bmjopen.bmj.com/site/about/guidelines.xhtml

## Background

Decisions relating to Do Not Attempt Cardiopulmonary Resuscitation (DNACPR) are complex and require tried and tested decision making processes as well as handover systems involving the patient, relevant others and a multidisciplinary healthcare team. Initiation of discussions of DNACPR is likely to occur if there is clinical evidence that CPR would be futile, that resulting harm would outweigh potential benefits or if a patient refuses CPR treatment. In England and Wales the Human Rights Act 1998[1] and the Mental Capacity Act 2005[2] provide the legal basis for DNACPR decision making. The Human Rights Act 1998[1] covers fundamental rights such as the right to life and the right to be free from inhuman and degrading treatment and The Mental Capacity Act 2005[2] works under the assumption that every adult is able to make their own decisions unless a mental capacity assessment shows otherwise. The latter sets out how to carry out an assessment of capacity, describes who can make decisions for people who lack capacity and provides a checklist to ensure that any decision taken on behalf of a person without capacity is in their best interest. Based on these laws, national guidelines[3] in England describe the context, setting and process for making informed decisions to omit CPR and provide a framework to support decisions relating to CPR. Nonetheless, recent high profile cases in the media suggest that issues exist in terms of DNACPR decision making in English health care settings claiming lack of consistency in approach across England. It is, therefore, of interest to understand the impact that the national guidelines have on local policies which, in the absence of a national DNACPR policy, determine local practice. By taking the view that current guidelines are informed primarily by ethical and legal considerations with little focus on the available research evidence there might be room for improvement in the practical guidance available. Furthermore, the guidelines provide general principles that require tailoring to local circumstances, which suggests that there may be room for interpretation of national guidelines when implemented into local policy. We therefore reviewed a random sample of local DNACPR policies from acute, community and ambulance services Trusts across England and mapped them against aspects from national guidelines to identify variation and consistencies between and within Trust types and inconsistencies in implementation of national guidelines.



For peer review only - http://bmjopen.bmj.com/site/about/guidelines.xhtml

## Methods

Using the NHS service directory,[4] we obtained care Trust lists and identified a random sample of 20 acute hospital Trusts for review. After mapping a further six Trusts were chosen to ensure geographical coverage. All 10 ambulance service (AS) Trusts and a random sample of 12 community health Trusts were further included. Freedom of information requests for adult DNACPR policies were sent to the sample of English acute Trusts, ambulance service Trusts and community Trusts. If a separate DNACPR policy was not available the Trust's resuscitation policy was requested. Follow-up e-mails were sent once if (1) the wrong policy was sent, (2) additional information was apparently available on reading of the policy or (3) the health Trust had not responded after two months.

Additional information given in the accompanying e-mails by the Trust on further policies and validity of the policy was considered in the data extraction process as was information on the back of the DNACPR form if provided.

A data extraction form was established using aspects important for DNACPR decision making from the joint statement by the Resuscitation Council (RC (UK)), the British Medical Association (BMA) and the Royal College of Nursing (RCN) on decisions relating to cardiopulmonary resuscitation.[3] The resulting extraction form was piloted by two reviewers and was subsequently adjusted. The final form included 26 questions on aspects taken from the national guidelines including documentation, ethical and legal issues, DNACPR decision makers and involvement of others in DNACPR decisions as well as practical considerations such as validity, review period and portability of decisions. Policies were then read in their entirety by one researcher (KF) and data extracted using the agreed form. Queries during the data extraction process were discussed and agreed with GDP. The first 30% of data extractions were checked by a second reviewer and confirmed as accurate (RF).

Data were synthesised quantitatively (documentation of DNACPR decisions, implementation of legal and ethical aspects, decision makers and involvement of others) and narratively (practical considerations). For the qualitative synthesis, policies were coded using descriptive coding. Main categories of topic areas were



For peer review only - http://bmjopen.bmj.com/site/about/guidelines.xhtml

identified by discussion through clustering of the codes. The major areas of interest included the validity, review and portability including handover of decisions.

## Results

A total of 48 local DNACPR policies were reviewed (100% response rate). The 26 acute Trusts consisted of six teaching hospitals, 19 district general hospitals and one specialist centre. Good geographical coverage of England was achieved with the reviewed policies from acute and community Trusts (N=12) (see Figure 1). All ten English AS were considered.

*Documentation*

The Trusts produced specific local documents referred to as policies (85%), procedures (6%) or guidelines (8%) (Table 1).There was variation in the terms used to describe resuscitation decisions with two thirds of Trusts referring to the term “DNACPR”, a quarter to “DNAR” and the remainders to “Not for CPR” or “Allow Natural Death”. Half the Trusts had reviewed the relevant policy/guideline/procedure (referred to from here under the uniform term “policy”) within the preceding 12 months (range 1-47 months). One quarter of policies were outside the review period set by the Trust while half indicated the expired policy was currently under active review. Three quarters of policies reported having undergone an equality and diversity assessment.

There was variation in how DNACPR decisions were recorded (Table 2). One quarter of Trusts used the RC (UK) DNACPR form or a modified version. As a general guide, modifications included adding or changing a couple of questions but required the general layout to be the same as the RC (UK) form while any more substantial changes would make the form bespoke. The majority of forms were paper forms (81%) with only 8% of Trusts using electronic versions. None of the forms required the patient to give consent. Health care professionals making the DNACPR decision were obligated to sign the form, which also had room for a countersignature by the consultant if a junior doctor initiated the decision which needs endorsement. In one specific case the form required that the doctor’s



For peer review only - http://bmjopen.bmj.com/site/about/guidelines.xhtml

signature must be witnessed by the registered nurse who was involved in the discussion on the DNACPR decision.

*Ethical and legal basis for DNACPR decisions*

The joint statement "Decisions relating to Cardiopulmonary Resuscitation" from the British Medical Association, the Resuscitation Council (UK) and the Royal College of Nursing was the most frequently cited source of national guidance for acute and community NHS Trusts. Reference to relevant legislation (Mental Capacity Act 89%, Human Rights Act, 78%) was common. General Medical Council guidelines were highlighted less frequently (50%). 9/10 Ambulance Trusts additionally cited the Joint Royal College Ambulance Liaison Committee.

Acute and community Trust policies universally correctly identified the circumstances in which a DNACPR decision could be considered (futility, overall benefit, patient refusal) and most clarified that the DNACPR decision related only to the act of resuscitation and did not apply to other aspects of care.

*Decision makers and involvement of others in DNACPR decisions*

All acute and community Trusts were responsible for primary DNACPR decisions whilst ambulance Trusts were not primary decision makers of lasting DNACPR decisions. The review identified variation in the grade of clinical staff authorised to make an initial DNACPR decision. Authority was delegated to senior nursing staff at 7 Trusts (1 limited to MacMillan Nurses, 3 for community decisions only, 3 appropriately qualified nurses) or junior medical staff (foundation year doctors (n=3), specialist trainee doctors (n=15), most senior available / any grade (n=6) or undefined / unclear (n=3)). Accountability for DNACPR decisions rested with consultants at all acute Trusts and the most senior clinician who may be a GP, consultant or nurse depending on circumstances at community Trusts. Few (8%) of Trusts mandated medical staff to discuss decisions with others within the multidisciplinary team although most (74%) recommended discussion, a fifth made no such recommendations.



For peer review only - http://bmjopen.bmj.com/site/about/guidelines.xhtml

Acute Trusts recommended staff to talk to patients and relatives in 100% of reviewed policies and community Trusts in 100% to patients and 92% to relatives. Guidance requiring clinical staff to assess the patient for capacity and guidance about when to consult a lasting power of attorney or independent mental capacity advocate occurred less commonly. Fifty percent of Trusts recommended the use of a patient information leaflet. Less than one in ten Trusts provided practical guidance on how to approach DNACPR decision making in different cultures (see electronic supplement for detailed information).

*Practical issues of DNACPR decision making: Validity, review and portability of DNACPR decisions*

The duration of time over which a DNACPR decision was considered valid varied widely. DNACPR decisions from acute Trusts ranged from valid indefinitely (54%) to valid for the duration of one hospital admission only (31%) and valid till a specific point in time (8%) or up until a specified review date (4%). Similarly, in community trusts validity ranged from valid indefinitely (42%) to valid up until a specified review date (42%). Two community Trusts had no information in the policies about the validity of decisions and the duration of validity in one acute Trust was unclear. There was similarly wide variation in Trusts' approach to reviewing DNACPR decisions with timings ranging from 24 hours to months (see electronic supplement for detailed information).

Ambulance services focused on the requirements to recognise a valid DNACPR decision from other organisations. The level of proof required ranged from original DNACPR form acceptable only (n=1) to photocopies with ink signature / legible signature accepted (n=2) and form not necessarily needed to be seen (n=1). Six policies did not specify the requirements on the level of proof of a valid DNACPR decision. Acceptable formats of DNACPR decisions varied considerably. Two Trusts recognised that DNACPR decisions come in a variety of formats and were willing to accept verbal and written DNACPR decisions (including letters, entry in patient notes and pro-forma). Another two Trusts accepted any kind of written decisions, while one Trust stated that a decision had to be presented on a DNACPR form. Three more Trusts required specific forms only. 7/10 policies specified details that staff need to

For peer review only

check to establish the validity of the document. These included the patient's details, the review date, a list of items if the decision is not on a pro-forma, and the completeness of transport specific sections on the DNACPR form.

The portability of DNACPR decisions between organisations and health care settings (community / acute care) was one of the greatest sources of inconsistency and variation. DNACPR decisions were portable in 13/26 acute Trusts and 8/12 community Trusts. An additional six acute Trusts' DNACPR decisions extended to include ambulance transport. The remaining acute Trusts (n=7) but only one community Trust had a system in place through which non-portable DNACPR decisions could be communicated between providers. The level of portability of DNACPR decisions in the remaining three community Trusts was unclear. The detail with which handover systems were described in the policies was generally greater in the community Trusts than the acute Trusts. An example of a clear, un-ambiguous pathway, describing transfer between and within acute and community care settings of a fully portable DNACPR form is provided in Box 1.

## Discussion

Our review of local DNACPR policies revealed that while some isolated aspects of the national guidelines were implemented consistently into local policy, there was generally huge variation between local policies in all areas of documentation, ethical and legal issues, the decision makers but first and foremost in the practical issues of DNACPR decision making, i.e. the validity, review and portability of decisions which greatly affect the interface between services. Some of the variation might be explained in part by the language used in the national guidelines leaving too much scope for interpretation while others reveal a lack of compliance with clear recommendations in the guidelines. While the guidelines focus on ethical and legal issues related to DNACPR decision making, there is less guidance on the more practical issues of DNACPR decisions. The national guidelines make no reference to the validity and do not provide guidance on the frequency of review of DNACPR decisions. There is also limited guidance on the portability of decisions, i.e. whether



For peer review only - http://bmjopen.bmj.com/site/about/guidelines.xhtml

decisions should be accepted automatically by health care providers outside the establishment of the primary decision maker and how the handover systems should be organised. The resulting variation in portability reflects the range of different systems that are in place across England of how DNACPR decisions are handled in English health care trusts.

While the abovementioned systems of handling DNACPR decisions confirm the variation in local policies of the different health care services there is also evidence of efforts of standardisation that are initiated by regional working groups rather than national leaders. Several regions in England have formulated unified policies with regional DNACPR forms to improve communication and handover across healthcare providers within one area. These initiatives are often sponsored by the relevant clinical commissioning groups and allow a consistent approach, easy recognition of forms and allow the decisions to cross the borders between primary and secondary healthcare. However, there is also evidence that signing up to regional policies and switching to unified forms is slow and challenging and it would be of interest to understand the barriers that hinder Trusts to go unified as these might be equivalent to barriers to the implementation of national guidelines or a possible future national policy.

The development of a national template or standardised policy to support clinicians and patients in decision making seems intuitively attractive. Such measure might improve consistency in approach and reduce policy variation. It would reduce the need for doctors moving between Trusts to learn new policies each time they go to a new Trust. Furthermore, it would allow the development of some generic learning materials on DNACPR decision making for clinicians and patients. However, the poor implementation of national guidelines and the slow and patchy recognition of regional policies might suggest that there are barriers to standardisation that need to be understood and overcome. Furthermore, there is a need to research and understand issues in the implementation and translation of local policy into local clinical practice as this would not be addressed by a standardised policy.

It is questionable that a national policy on the same lines as the national guidelines would achieve consistency in practice unless the practical issues of validity, review and portability are addressed. Local policies need more guidance on the practical



For peer review only - http://bmjopen.bmj.com/site/about/guidelines.xhtml

aspects to standardise approaches. This would hopefully have an impact on communication and handover of DNACPR decisions between healthcare providers. However, without evidence on which system works best it would be difficult to choose one system and subsequently mandate it for all health care Trusts. Another approach would be to support standardisation efforts and help unified policies to spread and award regional initiatives.

While it has been suggested that incorporating DNACPR forms into an overall treatment option form (Universal Form of Treatment Options - UFTO)[5] or into treatment escalation plans (TEP)[6] is associated with reduced patient harm[5] and improved communication with patients and relatives,[5 6] it is unlikely that an intervention that supports the initial decision making process will eliminate the problems of handover and communication between healthcare providers involved in the care of the patient.

Communication of DNACPR is a major issue. The guidelines covered the appropriateness of talking to patients or relevant others in detail, however, implementation into local policies was variable. Translation of policies into practice is a further issue which is evident but outside the scope of this review.

*National and international context*

The current national and international literature illustrates that challenges around guidance for DNACPR decision making is evidenced at all three levels, i.e. national policy or guidelines, local policy and local practice.

While our research revealed that in England national guidelines are implemented inconsistently into local policy, a recent survey of UK DNACPR forms suggested that changes in the national guidelines were the main driver for DNACPR form amendments as it was the most frequent response.[7] However, the majority of responses included a variety of other drivers for change (55/71) which seems to confirm that the influence of the guidelines is variable across England. More concerning is that in a survey of specialist registrars over one quarter of respondents was unable to recommend a document or guideline for DNACPR decision making.[8] The survey identified views of specialist registrars that there were no current guidelines that cover the complexities and difficulties of DNACPR decisions and that



For peer review only - http://bmjopen.bmj.com/site/about/guidelines.xhtml

the available guidelines contradicted themselves. This also seems to support our finding that national guidelines should be improved to provide clear recommendations in all aspects of DNACPR decision making.

Furthermore, national and international literature indicates that there are deficiencies in staff knowledge of local DNACPR policies[9] and relevant laws and regulations[10] which might be suggestive of a gap between policy and practice. For instance while our policy review suggested good compliance with national guidelines in terms of clarifying that the DNACPR decision related only to the act of resuscitation and did not apply to other aspects of care, Smith et al. (2006)[9] reported that about 15% of nurses and midwifes believed that antibiotics, physiotherapy and nasogastric feeding would be inappropriate for DNACPR patients. Additional knowledge gaps included uncertainties about which professional grade can make decisions; and health care professionals generally did not believe that informing the multidisciplinary team of DNACPR decisions was important.[9]

In contrast, facilitators for DNACPR decision making in the literature appear to focus on local practice. Imhof et al. (2011)[11] found that interdisciplinary decisions were important to avoid conflict and non-compliance. Their research stressed that integrating nurses' views and observations into the decision would lead to more successful outcomes in compliance with resuscitation decisions. Furthermore, they recommend that decision making should not be left to junior doctors because of the professional, human and relational expertise that is required.[11] However, involvement of nurses in the decision making process would require additional education of nurses to fill knowledge gaps in resuscitation legislation.[10]

Further facilitators to ease DNACPR decision making have been suggested. Myint et al. (2006)[8] suggested raising patient and public awareness of resuscitation and decisions around resuscitation. This is of interest as a review of articles in the lay-press revealed that newspapers in the UK portray an overoptimistic survival rate following cardiac arrest in the out-of hospital and in-hospital setting.[12] Further claims to improve DNACPR decision making were the implementation of a standard process in trust policies to record patients' wishes at admission[8] while Kim et al. (2007)[13] called for a systematic standardisation of DNACPR decisions in terms of standardised forms to improve end-of life decisions in the early stage of terminal



For peer review only - http://bmjopen.bmj.com/site/about/guidelines.xhtml

cancer patients. A recent systematic review confirmed these findings by ranking standardisation of DNACPR forms and structured changes to the decision making process for instance at the time of admission as the two most promising interventions identified in the literature that could improve DNACPR decision making.[14] Aspects for the successful implementation of a standardised approach to DNACPR decision making were reported in the pre-hospital setting in two states in the USA.[15] It showed that the introduction of a standardised form together with a core protocol at the same time as in-service training proved valuable for its success.[15] Approach of the Emergency Medical Services boards to gain approval also helped protect from the development of multiple forms as well as using unambiguous language and ensuring immunity from litigation. Finally, education of the public through news articles about the service was thought to be a particular strength of the programme.

In terms of policy and guidance published research is less specific in their recommendations. A survey of 298 Irish consultant physicians found that only 21% were aware of a formal resuscitation policy in their hospital which is why the authors called for a national policy for resuscitation decision making to facilitate more widespread formulation of local policies.[16]

Overall, the national and international literature tends to address issues around the initial decision making process including the involvement of nurses, standardisation and lack of awareness of legislation. However, there appears to be a lack of research addressing issues relating to the interface of healthcare settings for safe handover of DNACPR decisions.

*Strengths and limitations*

While this research reviewed only a sample of local policies, these covered all areas of England and met our objectives as this sample sufficiently identified huge variation and a worrying number of different systems of how Trusts deal with DNACPR decisions across England. Furthermore, the review included policies from different types of Trusts which was important as these concern health care providers with different responsibilities. The ambulance services for instance are not primary decision makers of lasting DNACPR decisions and are therefore more concerned with the handover and checking the validity of decisions. This relates to lasting



For peer review only - http://bmjopen.bmj.com/site/about/guidelines.xhtml

DNACPR decisions only. AS staff attending patients in cardiac arrest still have to make immediate but informed decisions on whether to commence CPR or when to stop CPR. However, this research concentrated on lasting DNACPR decisions that AS face when accepting a DNACPR patient from acute Trusts or the community.

Community Trusts are an assemblage of diverse health care providers. Their concern, therefore, concentrates on the handover, communication and consistent documentation of decisions within the Trust. This might explain why community services tended to describe handover systems in more detail than acute Trusts and why the proportion of portable decision and unified policies was greater among community than acute Trusts.

As our research only addressed the implementation of national guidelines into local policies it does not highlight shortfalls in the implementation of national and local guidance into local practice. There is a need to investigate issues and possible solutions to improve uptake of guidance into local practice.

## Conclusions

This review identified significant variation in English NHS Trusts approaches to DNACPR decision making. Gaps identified included practical guidance around when and how to communicate DNACPR decisions and in relation to the portability / transferability of decisions between healthcare settings. There is a need for greater consistency in Trusts approaches to DNACPR decision making.

## Acknowledgement

This project was funded by the National Institute for Health Research [Health Service Research and Delivery Programme, project number 12/5001/55]. The views and opinions expressed therein are those of the authors and do not necessarily reflect those of the [Health Service Research and Delivery Programme], NIHR, NHS or the Department of Health.



For peer review only - http://bmjopen.bmj.com/site/about/guidelines.xhtml

GDP is a Director of Research for the Intensive Care Foundation.

## References

1. Great Britain. Human Rights Act 1998. [online]. http://www.legislation.gov.uk/ukpga/1998/42/contents (accessed 16 June 2014).
2. Department for Constitutional Affairs. Mental capacity Act 2005: Code of practice. https://www.justice.gov.uk/downloads/protecting-the-vulnerable/mca/mca-code-practice-0509.pdf (accessed 22 May 2014).
3. British Medical Association, Resuscitation Council (UK), and Royal College of Nursing. Decisions relating to cardiopulmonary resuscitation: A joint statement from the British Medical Association, the Resuscitation Council (UK) and the Royal College of Nursing 2007. http://www.resus.org.uk/pages/dnar.pdf (accessed 16 June 2014).
4. National Health Service. NHS Choices. Service Directories Authorities and Trusts. http://www.nhs.uk/ServiceDirectories/Pages/AcuteTrustListing.aspx (accessed 16 June 2014).
5. Fritz Z, Malyon A, Frankau JM, et al. The Universal Form of Treatment Options (UFTO) as an alternative to Do Not Attempt Cardiopulmonary Resuscitation (DNACPR) orders: a mixed methods evaluation of the effects on clinical practice and patient care. PloS one 2013;**8**(9):e70977.
6. Obolensky L, Clark T, Matthew G, et al. A patient and relative centred evaluation of treatment escalation plans: a replacement for the do-not-resuscitate process. Journal of medical ethics 2010;**36**(9):518-20.
7. Clements M, Fuld J, Fritz Z. Documentation of resuscitation decision-making: a survey of practice in the United Kingdom. Resuscitation 2014;**85**(5):606-11.
8. Myint PK, Miles S, Halliday DA, et al. Experiences and views of specialist registrars in geriatric medicine on 'do not attempt resuscitation' decisions: a sea of uncertainty? QJM : monthly journal of the Association of Physicians 2006;**99**(10):691-700.
9. Smith GB, Poplett N, Williams D. Staff awareness of a 'Do Not Attempt Resuscitation' policy in a District General Hospital. Resuscitation 2005;**65**(2):159-63.
10. Saevareid TJ, Balandin S. Nurses' perceptions of attempting cardiopulmonary resuscitation on oldest old patients. Journal of advanced nursing 2011;**67**(8):1739-48.
11. Imhof L, Mahrer-Imhof R, Janisch C, et al. Do not attempt resuscitation: the importance of consensual decisions. Swiss medical weekly 2011;**141**:w13157.
12. Field RA, Soar J, Nolan JP, et al. Epidemiology and outcome of cardiac arrests reported in the lay-press: an observational study. Journal of the Royal Society of Medicine 2011;**104**(12):525-31.
13. Kim do Y, Lee KE, Nam EM, et al. Do-not-resuscitate orders for terminal patients with cancer in teaching hospitals of Korea. Journal of palliative medicine 2007;**10**(5):1153-8.
14. Field RA, Fritz Z, Baker A, et al. Systematic Review of Interventions to Improve Appropriate Use and Outcomes Associated with Do-Not-Attempt-Cardiopulmonary-Resuscitation Decisions. Resuscitation 2014 in press 2014



For peer review only - http://bmjopen.bmj.com/site/about/guidelines.xhtml

15. Sosna DP, Christopher M, Pesto MM, et al. Implementation strategies for a do-not-resuscitate program in the prehospital setting. Annals of emergency medicine 1994;**23**(5):1042-6
16. Butler MW, Saaidin N, Sheikh AA, et al. Dissatisfaction with Do Not Attempt Resuscitation Orders: A nationwide study of Irish consultant physician practices. Irish medical journal 2006;**99**(7):208-10

For peer review only

**Figures and tables**



For peer review only - http://bmjopen.bmj.com/site/about/guidelines.xhtml

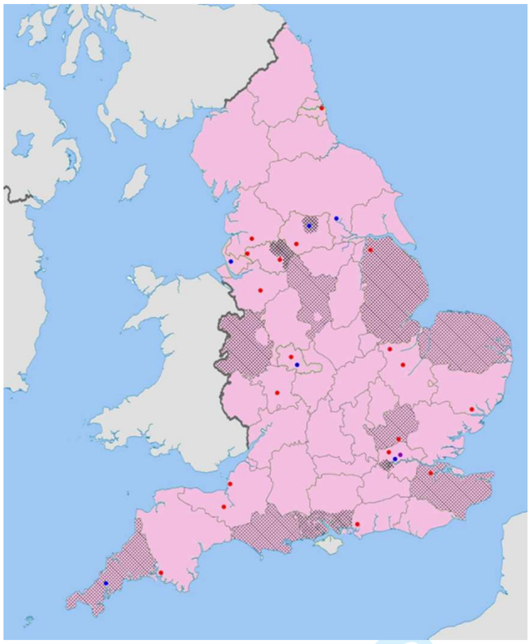

**Figure 1** Overview and geographic coverage of local Trust DNACPR policies included in the review
(• Teaching hospital; • District general hospital; • Specialist hospital; ▩ Community healthcare Trust)

**Table 1** Overview of variation in type of local DNACPR policies by Trust type

| Policy type item | Acute Trusts (n=26), n(%) | Community Trusts (n=12), n (%) | AS Trusts (n=10), n (%) | Total, N=48, n (%) |
|---|---|---|---|---|
| TYPE OF DOCUMENT | | | | |
| Policy | 22 (85%) | 12 (100%) | 7 (70%) | 41 (85%) |
| Procedure | 2 (8%) | 0 | 1 (10%) | 3 (6%) |
| Guideline | 2 (8%) | 0 | 2 (20%) | 4 (8%) |
| TERMINOLOGY | | | | |
| DNAR | 6 (23%) | 3 (25%) | 3 (30%) | 12 (25%) |
| DNACPR | 16 (62%) | 8 (67%) | 7 (70%) | 31 (65%) |
| Not for CPR | 3 (12%) | 0 | 0 | 3 (6%) |
| AND | 1 (4%) | 2 (17%)* | 0 | 3 (6%) |
| VISIBILITY | | | | |
| Stand alone | 21 (81%) | 9 (75%) | 3 (30%) | 33 (69%) |
| Integrated into resuscitation policy | 5 (19%) | 3 (25%) | 3 (30%) | 11 (23%) |
| Mentioned in other policies | 0 | 0 | 4 (40%) | 4 (8%) |
| COVERAGE | | | | |
| Local | 23 (88%) | 9 (75%) | 8 (80%) | 40 (83%) |
| Regional | 3 (12%) | 3 (25%) | 2 (20%) | 8 (17%) |

*Column does not add up to 12 because one Trusts consciously and consistently referred to DNACPR/AND throughout the policy



For peer review only - http://bmjopen.bmj.com/site/about/guidelines.xhtml

(Abbreviations: AS Ambulance service, DNAR do not attempt resuscitation, DNACPR do not attempt cardiopulmonary resuscitation, CPR cardiopulmonary resuscitation, AND allow natural death)

**Table 2** Overview of adoption of RC (UK) DNACPR forms and type of form by acute, community and AS Trusts

| DNACPR form | Acute Trusts (n=26), n(%) | Community Trusts (n=12), n (%) | AS Trusts (n=10), n (%) | Total, N=48, n (%) |
|---|---|---|---|---|
| RC (UK) | 2 (7.8%) | 3 (25%) | 0 | 5 (10.4%) |
| Modified | 4 (15.4%) | 3 (25%) | 1 (10%) | 8 (16.7%) |
| Bespoke | 16 (61.5%) | 4 (33.3%) | 4 (40%) | 24 (50%) |
| Form not sent | 4 (15.4%) | 2 (16.6%) | 5 (50%) | 11 (22.9%) |
| Type of form* | | | | |
| Paper | 24 (92.3%) | 10 (83.3%) | 5 (50%) | 39 (81.3%) |
| Electronic | 1 (3.8%) | 0 | 0 | 1 (2.1%) |
| Both | 1 (3.8%) | 2 (16.6%) | 0 | 3 (6.3%) |

*Insufficient information was available in policies from 5 Trusts for this question
(Abbreviations: RC (UK) Resuscitation Council (UK), DNACPR do not attempt cardiopulmonary resuscitation, AS ambulance service)

**Box 1** Example of clear pathway for handover of DNACPR decision from one acute trust

Review the appropriateness of the DNACPR decision before discharge

If on review the DNACPR is still considered appropriate carry decision over to the patient's care setting/home on discharge including the following considerations:

- Liaise with the patient's GP and identify an agreed, appropriate community review date (ideally within 24 hours), which should be documented on the DNACPR form
- Send the original DNACPR form (top white copy) with a review date in place with the patient
- Communicate and discuss sensitively with the patient
- Leave the yellow copy (second copy) in the front of the patient's medical hospital notes facilitating early consideration of resuscitation issues on any potential subsequent readmission

Communication with Ambulance Service and / or ongoing care setting must take place

Before Ambulance transfer complete appropriate section on the DNACPR form

When booking transport for DNACPR patient from acute Trust fax DNACPR form to ambulance control Contact Centre

Within community settings, hand DNACPR forms directly to the attending crew on arrival or fax to AS Contact Centre

The DNACPR status must also be recorded by the attending AS crew on the AS Patient Report Form



For peer review only - http://bmjopen.bmj.com/site/about/guidelines.xhtml

## Electronic supplementary material

Table E1 Compliance with the requirement to refer to relevant laws / guidelines in local policies by acute, community and AS Trusts

| Item from national guidelines | Acute Trusts (n=26), n (%) | Community Trusts (n=12), n (%) | AS Trusts (n=10), n (%) | Total, N=48, n (%) |
|---|---|---|---|---|
| Has an equality and diversity assessment been carried out? | 17 (65.4%) | 10 (83.3%) | 9 (90%) | 36 (75%) |
| Does the policy describe all three situations (futility, burden, refusal) in which DNACPR decisions are appropriate? | 26 (100%) | 12 (100%) | 6 (60%) | 44 (91.7%) |
| Does the policy require staff to assess patient capacity? | 15 (57.7%) | 9 (75%) | 1 (10%) | 25 (52.1%) |
| Does the policy clarify the relationship between DNACPR and other aspects of care? | 25 (96.2%) | 11 (91.7%) | 9 (90%) | 45 (93.8%) |
| Does the policy contain the RCUK flow diagram to guide DNACPR decisions yes or modified? | 20 (41.7%) | 10 (83.3%) | 3 (30%) | 33 (68.8%) |
| Does the policy contain guidance relating to cultural differences? | 3 (11.5%) | 1 (8.3%) | 0 | 4 (8.3%) |
| Does the policy refer to a patient information leaflet for DNACPR? yes [RC (UK)] | 12 [1] (46.2% [3.8%]) | 9 [0] (75%) | 1 (10%) | 22 [1] (45.8% [2.1%]) |
| Does the policy contain information on audit? | 26 (100%) | 12 (100) | 9 (90%) | 47 (97.9%) |

(Abbreviations: AS ambulance service, DNACPR do not attempt cardiopulmonary resuscitation, RC (UK) Resuscitation Council (UK))

Table E2 Compliance with the requirement to consult patients and relevant others by acute, community and AS Trusts

| Does the policy tell staff when to consult / inform patients or relevant others? | Acute Trusts (n=26), n (%) | Community Trusts (n=12), n (%) | AS Trusts (n=10), n (%) | Total, N=48, n (%) |
|---|---|---|---|---|
| Patients | 26 (100%) | 12 (100%) | 6 (60%) | 44 (91.7%) |



For peer review only - http://bmjopen.bmj.com/site/about/guidelines.xhtml

| | | | | |
|---|---|---|---|---|
| LPA | 20 (41.7%) | 11 (91.7%) | 3 (30%) | 34 (70.8%) |
| Relatives | 26 (100%) | 11 (91.7%) | 5 (50%) | 42 (87.5%) |
| IMCA | 18 (69.2%) | 8 (66.7%) | 2 (20%) | 28 (58.3%) |

(Abbreviations: AS ambulance service, LPA lasting power of attorney, IMCA independent mental capacity advocate)

Table E3 Compliance with the requirement to consult a multidisciplinary team for decision a making by acute and community Trusts

| **Consultation of multidisciplinary team** | **Acute Trusts (n=26), n (%)** | **Community Trusts (n=12), n (%)** | **Total, N=38, n (%)** |
|---|---|---|---|
| Must | 1 (3.8%) | 2 (16.6%) | 3 (7.9%) |
| Should | 22 (84.6%) | 6 (50%) | 28 (73.7%) |
| Not mentioned | 3 (11.5%) | 4 (33.3%) | 7 (18.4%) |

**Policies on review of DNACPR decisions**

Policies on review ranged from regular review required with or without specification of a review date to regular review not recommended. The majority of policies included specific prompts that required clinicians to initiate review. These prompts included admission / readmission, transfer / discharge, surgery or anaesthesia and the change of the patient's condition or a change of heart.

A review period was given in 7/26 (27%) policies from acute trusts which ranged from 24 hours to 6 months. Six policies (23%) recommended regular review but did not specify a particular time period, while four (15%) policies did not recommend regular review. One stated that review was not required and eight (31%) did not mention regular review. Specific prompts for review were admission/readmission (n=17), transfer/discharge (n=16) and surgery/anaesthesia (n=16). Clinical discretion would prompt a review in 22/26 (85%) policies. And a change in the patient's condition or patient's expressed wishes as a reason for review was mentioned in 24/26 (92%) policies.

Similarly, regular review was mentioned in 5/12 policies from community trusts, which ranged from weekly (n=2) to 6 monthly (n=1) and not specified (n=2). One trust did not recommend regular review. The frequency of the review period was determined by clinical judgment and the change in patient status / patient change of mind in the majority of policies (8/12 and 11/12 respectively). Specific situations that



For peer review only - http://bmjopen.bmj.com/site/about/guidelines.xhtml

prompted review were admission/readmission (7/12), transfer/discharge (9/12) and surgery/anaesthesia (4/12).

5/10 policies from ambulance services did not mention anything on review. 3/10 policies stated that if a review date is specified on the form, AS staff need to check whether the decision is within the review period. 5/10 policies stated that decisions without review date including decisions for terminal ill patients should be considered as indefinite. For other patients regular review is recommended in three of these five policies, while one policy does not recommend regular review. In 5/10 policies review is required at clinical discretion. Situations that should prompt a review were change in the patient's condition or change in expressed wishes (n=3), admission / readmission (n=1) and transfer / discharge (n=1). One policy recognised that review dates on forms will vary across the region according to different local policies. And one policy included a form that stated that no decision should be regarded as indefinite and that the review period must be no longer than 3 months.

For peer review only

**Electronic supplementary material**

Table E1 Compliance with the requirement to refer to relevant laws / guidelines in local policies by acute, community and AS Trusts

| Item from national guidelines | Acute Trusts (n=26), n (%) | Community Trusts (n=12), n (%) | AS Trusts (n=10), n (%) | Total, N=48, n (%) |
|---|---|---|---|---|
| Has an equality and diversity assessment been carried out? | 17 (65.4%) | 10 (83.3%) | 9 (90%) | 36 (75%) |
| Does the policy describe all three situations (futility, burden, refusal) in which DNACPR decisions are appropriate? | 26 (100%) | 12 (100%) | 6 (60%) | 44 (91.7%) |
| Does the policy require staff to assess patient capacity? | 15 (57.7%) | 9 (75%) | 1 (10%) | 25 (52.1%) |
| Does the policy clarify the relationship between DNACPR and other aspects of care? | 25 (96.2%) | 11 (91.7%) | 9 (90%) | 45 (93.8%) |
| Does the policy contain the RCUK flow diagram to guide DNACPR decisions yes or modified? | 20 (41.7%) | 10 (83.3%) | 3 (30%) | 33 (68.8%) |
| Does the policy contain guidance relating to cultural differences? | 3 (11.5%) | 1 (8.3%) | 0 | 4 (8.3%) |
| Does the policy refer to a patient information leaflet for DNACPR? yes [RC (UK)] | 12 [1] (46.2% [3.8%]) | 9 [0] (75%) | 1 (10%) | 22 [1] (45.8% [2.1%]) |
| Does the policy contain information on audit? | 26 (100%) | 12 (100) | 9 (90%) | 47 (97.9%) |

(Abbreviations: AS ambulance service, DNACPR do not attempt cardiopulmonary resuscitation, RC (UK) Resuscitation Council (UK))



For peer review only - http://bmjopen.bmj.com/site/about/guidelines.xhtml

Table E2 Compliance with the requirement to consult patients and relevant others by acute, community and AS Trusts

| Does the policy tell staff when to consult / inform patients or relevant others? | **Acute Trusts (n=26), n (%)** | **Community Trusts (n=12), n (%)** | **AS Trusts (n=10), n (%)** | **Total, N=48, n (%)** |
|---|---|---|---|---|
| Patients | 26 (100%) | 12 (100%) | 6 (60%) | 44 (91.7%) |
| LPA | 20 (41.7%) | 11 (91.7%) | 3 (30%) | 34 (70.8%) |
| Relatives | 26 (100%) | 11 (91.7%) | 5 (50%) | 42 (87.5%) |
| IMCA | 18 (69.2%) | 8 (66.7%) | 2 (20%) | 28 (58.3%) |

(Abbreviations: AS ambulance service, LPA lasting power of attorney, IMCA independent mental capacity advocate)

Table E3 Compliance with the requirement to consult a multidisciplinary team for decision a making by acute and community Trusts

| **Consultation of multidisciplinary team** | **Acute Trusts (n=26), n (%)** | **Community Trusts (n=12), n (%)** | **Total, N=38, n (%)** |
|---|---|---|---|
| Must | 1 (3.8%) | 2 (16.6%) | 3 (7.9%) |
| Should | 22 (84.6%) | 6 (50%) | 28 (73.7%) |
| Not mentioned | 3 (11.5%) | 4 (33.3%) | 7 (18.4%) |

**Policies on review of DNACPR decisions**

Policies on review ranged from regular review required with or without specification of a review date to regular review not recommended. The majority of policies included specific prompts that required clinicians to initiate review. These prompts included admission / readmission, transfer / discharge, surgery or anaesthesia and the change of the patient’s condition or a change of heart.

A review period was given in 7/26 (27%) policies from acute trusts which ranged from 24 hours to 6 months. Six policies (23%) recommended regular review but did not specify a particular time period, while four (15%) policies did not recommend



For peer review only - http://bmjopen.bmj.com/site/about/guidelines.xhtml

regular review. One stated that review was not required and eight (31%) did not mention regular review. Specific prompts for review were admission/readmission (n=17), transfer/discharge (n=16) and surgery/anaesthesia (n=16). Clinical discretion would prompt a review in 22/26 (85%) policies. And a change in the patient's condition or patient's expressed wishes as a reason for review was mentioned in 24/26 (92%) policies.

Similarly, regular review was mentioned in 5/12 policies from community trusts, which ranged from weekly (n=2) to 6 monthly (n=1) and not specified (n=2). One trust did not recommend regular review. The frequency of the review period was determined by clinical judgment and the change in patient status / patient change of mind in the majority of policies (8/12 and 11/12 respectively). Specific situations that prompted review were admission/readmission (7/12), transfer/discharge (9/12) and surgery/anaesthesia (4/12).

5/10 policies from ambulance services did not mention anything on review. 3/10 policies stated that if a review date is specified on the form, AS staff need to check whether the decision is within the review period. 5/10 policies stated that decisions without review date including decisions for terminal ill patients should be considered as indefinite. For other patients regular review is recommended in three of these five policies, while one policy does not recommend regular review. In 5/10 policies review is required at clinical discretion. Situations that should prompt a review were change in the patient's condition or change in expressed wishes (n=3), admission / readmission (n=1) and transfer / discharge (n=1). One policy recognised that review dates on forms will vary across the region according to different local policies. And one policy included a form that stated that no decision should be regarded as indefinite and that the review period must be no longer than 3 months.

For peer review only

BMJ Open

# Variation in local Trust Do Not Attempt Cardiopulmonary Resuscitation policies: a review of 48 English health care trusts


| | |
|---|---|
| Journal: | *BMJ Open* |
| Manuscript ID: | bmjopen-2014-006517.R1 |
| Article Type: | Research |
| Date Submitted by the Author: | 03-Oct-2014 |
| Complete List of Authors: | Freeman, Karoline; Warwick Medical School,<br>Field, Richard; Heartlands Hospital,<br>Perkins, Gavin; University of Warwick, Clinical Trials Unit |
| <b>Primary Subject Heading</b>: | Health policy |
| Secondary Subject Heading: | Emergency medicine, Communication, Health policy, Intensive care, Palliative care |
| Keywords: | INTERNAL MEDICINE, MEDICAL ETHICS, PALLIATIVE CARE, Adult intensive & critical care < INTENSIVE & CRITICAL CARE, Health policy < HEALTH SERVICES ADMINISTRATION & MANAGEMENT, Clinical governance < HEALTH SERVICES ADMINISTRATION & MANAGEMENT |
| | |


SCHOLARONE™
Manuscripts

For peer review only

# Variation in local Trust Do Not Attempt Cardiopulmonary Resuscitation policies: a review of 48 English health care trusts

Karoline Freeman[1], Richard A. Field[1,2], Gavin D. Perkins[1,2]

[1] Division of Health Sciences, Warwick Medical School, University of Warwick, Coventry, CV4 7AL, UK

[2] Heart of England NHS Foundation Trust, Bordesley Green East, Birmingham, B9 5SS, UK

**Corresponding author:**

Gavin D Perkins

g.d.perkins@warwick.ac.uk

Tel: 02476 50925

Fax: 024 7615 1136

**Keywords:**

Cardiopulmonary resuscitation

Do not attempt resuscitation

Do not attempt cardiopulmonary resuscitation

End of Life Care

Heart arrest

**Word count:** 4436

For peer review only

## Abstract

**Objectives:** To explore do not attempt cardiopulmonary resuscitation (DNACPR) policies from English acute, community and ambulance service Trusts for evidence of consistency and variation in implementation of national guidelines between healthcare organisations.

**Setting:** Acute, community or ambulance NHS Trusts in England.

**Participants:** 48 NHS Trusts.

**Interventions:** Freedom of information requests for adult DNACPR policies were sent to a random sample of Trusts.

**Outcomes:** DNACPR policies were assessed on aspects identified from national guidelines including documentation, ethical and legal issues, decision makers and involvement of others in DNACPR decisions as well as practical considerations such as validity, review and portability of decisions.

**Results:** Policies from 26 acute, 12 community and 10 ambulance service Trusts were reviewed. There was variation in terminology used (85% described documents as policies, 6% procedures and 8% guidelines). Only one quarter of Trusts used the recommended Resuscitation Council (UK) record form (or a modification of the form). There was variation in the terminology used which included DNAR, DNACPR, Not for CPR and AND (allow natural death). Accountability for DNACPR decisions rested with consultants at all acute Trusts and the most senior clinician at community Trusts. Most Trusts (74%) recommended discussion of decisions with a multi-disciplinary team. Compliance with guidance requiring clinical staff to assess the patient for capacity and when to consult a lasting power of attorney or independent mental capacity advocate occurred less commonly. There was wide variation in the duration of time over which a DNACPR decision was considered valid as well as in the Trusts' approach to reviewing DNACPR decisions. The level of portability of DNACPR decisions between health care organisations was one of the greatest sources of variation.




For peer review only - http://bmjopen.bmj.com/site/about/guidelines.xhtml

**Conclusions:** There is significant variation in the translation of the national DNACPR guidelines into English health care Trusts' DNACPR policies.

For peer review only



For peer review only - http://bmjopen.bmj.com/site/about/guidelines.xhtml

## Strengths and limitations of this study

- Key areas for strengthening current approaches were identified and included improving consistency for recording decisions and ensuring decisions are transferable between healthcare settings
- Whether improving consistency in DNACPR policies will translate to improved implementation into practice and patient experience requires further study
- The findings require confirmation in other settings

For peer review only



For peer review only - http://bmjopen.bmj.com/site/about/guidelines.xhtml

## Background

Decisions relating to Do Not Attempt Cardiopulmonary Resuscitation (DNACPR) are complex and require tried and tested decision making processes as well as handover systems involving the patient, relevant others and a multidisciplinary healthcare team. Initiation of discussions of DNACPR is likely to occur if there is clinical evidence that CPR would be futile, that resulting harm would outweigh potential benefits or if a patient refuses CPR treatment. DNACPR decisions do not involve decisions about any other acute, life-saving treatments. In England the Human Rights Act 1998[1] and the Mental Capacity Act 2005[2] provide the legal basis for DNACPR decision making. The Human Rights Act 1998[1] covers fundamental rights such as the right to life, the right to be free from inhuman and degrading treatment and the right to hold opinions and to receive information. The Mental Capacity Act 2005[2] works under the assumption that every adult is able to make their own decisions unless a mental capacity assessment shows otherwise. The latter sets out how to carry out an assessment of capacity, describes who can make decisions for people who lack capacity and provides a checklist to ensure that any decision taken on behalf of a person without capacity is in their best interest. Based on these laws, national guidelines[3] in England describe the context, setting and process for making informed decisions to omit CPR and provide a framework to support decisions relating to CPR. Nonetheless, recent high profile cases in the media suggest that issues exist in terms of DNACPR decision making in English health care settings claiming lack of consistency in approach across England. It is, therefore, of interest to understand the impact that the national guidelines have on local policies which, in the absence of a national DNACPR policy, determine local practice. By taking the view that current guidelines are informed primarily by ethical and legal considerations with little focus on the available research evidence there might be room for improvement in the practical guidance available. Furthermore, the guidelines provide general principles that require tailoring to local circumstances, which suggests that there may be room for interpretation of national guidelines when implemented into local policy. We therefore reviewed a random sample of local DNACPR policies from acute, community and ambulance services Trusts across England and mapped them against aspects from national guidelines to identify variation and consistencies between and within Trust types and inconsistencies in implementation of national guidelines.



For peer review only - http://bmjopen.bmj.com/site/about/guidelines.xhtml

For peer review only

## Methods

Using the NHS service directory,[4] we obtained NHS care Trust lists of acute, community and AS service Trust. We identified a random sample of 20 acute hospital Trusts for review: Trust lists were numbered and, using a random number generator in EXCEL (using the RAND and INDEX functions), a random list without duplication was created. The first 20 trusts on this list were included in the review. After mapping a further six Trusts were chosen to ensure geographical coverage. All 10 ambulance service (AS) Trusts and a random sample of 12 community health Trusts were further included using the same method as described for acute Trusts. Freedom of information requests for adult DNACPR policies were sent to the sample of English acute Trusts, ambulance service Trusts and community Trusts. If a separate DNACPR policy was not available the Trust's resuscitation policy was requested. Follow-up e-mails were sent once if (1) the wrong policy was sent, (2) additional information was apparently available on reading of the policy or (3) the health Trust had not responded after two months.

Additional information given in the accompanying e-mails by the Trust on further policies and validity of the policy was considered in the data extraction process as was information on the back of the DNACPR form if provided.

A data extraction form was established using aspects from the joint statement by the Resuscitation Council (RC (UK)), the British Medical Association (BMA) and the Royal College of Nursing (RCN) on decisions relating to cardiopulmonary resuscitation.[3] Aspects were chosen that were deemed important for DNACPR decision making by the expert advisory group including national leads in DNACPR guidelines, practicing clinicians in acute, community and palliative care as well as patient representatives.  The resulting extraction form was piloted by two reviewers and was subsequently adjusted. The final form included 26 questions on aspects taken from the national guidelines including documentation, ethical and legal issues, DNACPR decision makers and involvement of others in DNACPR decisions as well as practical considerations such as validity, review period and portability of decisions (see electronic supplement for data extraction table E1). Policies were then read in their entirety by one researcher (KF) and data extracted using the agreed form.



For peer review only - http://bmjopen.bmj.com/site/about/guidelines.xhtml

Queries during the data extraction process were discussed and agreed with GDP. 30% of data extractions were checked by a second reviewer (RF) and confirmed as accurate, therefore further checking was not undertaken.

Data were synthesised quantitatively. Quantitative synthesis involved the reporting of frequencies of responses per question in tables for the paragraphs on documentation of DNCAPR decisions, implementation of legal and ethical aspects, decision makers and involvement of others. These covered the questions that involved answers chosen from a list of limited options (e.g. yes / no / uncertain). For the questions concerning the validity, review and portability of decisions the review of policies identified substantial variation in local policy documents. Comprehensive data extraction was undertaken to capture all identified variation in the policies in these three areas in separate documents using descriptive coding. The main categories of the topic areas were then identified by discussion through clustering of the codes between the three authors. The categories were presented to the project and advisory groups for consensus and subsequently formed the basis for possible responses to the questions on the data extraction form. In order to retain the breadth and depth of the variation within the categories the data presented in the paragraph 'practical considerations' was synthesised narratively. The major areas of interest included the validity, review and portability including handover of decisions.

## Results

A total of 48 local DNACPR policies were reviewed (100% response rate). The 26 acute Trusts consisted of six teaching hospitals, 19 district general hospitals and one specialist centre. Good geographical coverage of England was achieved with the reviewed policies from 26/156 acute and 12/24 community Trusts ~~(N=12)~~ (see Figure 1). All ten English AS were considered.

*Documentation*

The Trusts produced specific local documents referred to as policies (85%), procedures (6%) or guidelines (8%) (Table 1).There was variation in the terms used to describe resuscitation decisions. While the national guidelines recommend the



For peer review only - http://bmjopen.bmj.com/site/about/guidelines.xhtml

use of the phrase ‘do not attempt cardiopulmonary resuscitation’ to avoid confusion,[3] two thirds of Trusts referred to the term “DNACPR”, a quarter to “DNAR” and the remainders to “Not for CPR” or “Allow Natural Death”. Half the Trusts had reviewed the relevant policy/guideline/procedure (referred to from here under the uniform term “policy”) within the preceding 12 months (range 1-47 months). One quarter of policies were outside the review period set by the Trust while half indicated the expired policy was currently under active review. Three quarters of policies reported having undergone an equality and diversity assessment.

There was variation in how DNACPR decisions were recorded (Table 2). The guidelines clearly state that there is some benefit in using a standardised form and recommend the adoption of the RC(UK) model form.[3] In contrast, only one quarter of Trusts used the RC (UK) DNACPR form or a modified version. As a general guide, modifications included adding or changing a couple of questions but required the general layout to be the same as the RC (UK) form while any more substantial changes would make the form bespoke. The majority of forms were paper forms (81%) with only 8% of Trusts using electronic versions. Health care professionals making the DNACPR decision were obligated to sign the form, which also had room for a countersignature by the consultant if a junior doctor initiated the decision which needs endorsement. In one specific case the form required that the doctor’s signature must be witnessed by the registered nurse who was involved in the discussion on the DNACPR decision.

*Ethical and legal basis for DNACPR decisions*

The joint statement “Decisions relating to Cardiopulmonary Resuscitation” from the British Medical Association, the Resuscitation Council (UK)and the Royal College of Nursing was the most frequently cited source of national guidance for acute and community NHS Trusts. Reference to relevant legislation (Mental Capacity Act 89%, Human Rights Act, 78%) was common. General Medical Council guidelines were highlighted less frequently (50%). 9/10 Ambulance Trusts additionally cited the Joint Royal College Ambulance Liaison Committee.



For peer review only - http://bmjopen.bmj.com/site/about/guidelines.xhtml

The national guidance that DNACPR decisions may be made on the basis of futility, overall benefit or patient refusal was incorporated into all acute and community Trust policies. Most policies clarified that the DNACPR decision related only to the act of resuscitation and did not apply to other aspects of care.

*Decision makers and involvement of others in DNACPR decisions*

All acute and community Trusts were responsible for primary DNACPR decisions whilst ambulance Trusts were not primary decision makers of lasting DNACPR decisions. The review identified variation in the grade of clinical staff authorised to make an initial DNACPR decision. Authority was delegated to senior nursing staff at 7 Trusts (1 limited to MacMillan Nurses, 3 for community decisions only, 3 appropriately qualified nurses) or junior medical staff (foundation year doctors (n=3), specialist trainee doctors (n=15), most senior available / any grade (n=6) or undefined / unclear (n=3)). Accountability for DNACPR decisions rested with consultants at all acute Trusts and the most senior clinician who may be a GP, consultant or nurse depending on circumstances at community Trusts. This is in line with the national guidelines which state that local policy should define who the most senior person in charge of the DNACPR decision is. However, the guidelines do not make any recommendations on the staff grade that can initiate decisions.[3] Few (8%) of the Trusts mandated medical staff to discuss decisions with others within the multidisciplinary team although most (74%) recommended discussion, a fifth made no such recommendations.

Acute Trusts recommended staff to talk to patients and relatives in 100% of reviewed policies and community Trusts in 100% to patients and 92% to relatives. Guidance requiring clinical staff to assess the patient for capacity and guidance about when to consult a lasting power of attorney or independent mental capacity advocate occurred less commonly. Fifty percent of Trusts recommended the use of a patient information leaflet. Less than one in ten Trusts provided practical guidance on how to approach DNACPR decision making in different cultures. (See electronic supplement Tables E2 to E4 for more information on these aspects.)



For peer review only - http://bmjopen.bmj.com/site/about/guidelines.xhtml

*Practical issues of DNACPR decision making: Validity, review and portability of DNACPR decisions*

The national guidelines provide no guidance on how long DNACPR decisions should be valid and make no reference to the validity of decisions other than that the validity of decisions needs to be confirmed by receiving health care providers before accepting formal DNACPR decisions.[3] The duration of time over which a DNACPR decision was considered valid varied widely across local policies. DNACPR decisions from acute Trusts ranged from valid indefinitely (54%) to valid for the duration of one hospital admission only (31%) and valid till a specific point in time (8%) or up until a specified review date (4%). Similarly, in community trusts validity ranged from valid indefinitely (42%) to valid up until a specified review date (42%). Two community Trusts had no information in the policies about the validity of decisions and the duration of validity in one acute Trust was unclear. There was similarly wide variation in Trusts' approach to reviewing DNACPR decisions with timings ranging from 24 hours to months. (See electronic supplement for detailed information.) In terms of review the guidelines state that decisions must be reviewed regularly and that the frequency of review should be determined by the health professional in charge.[3]

Ambulance services focused on the requirements to recognise a valid DNACPR decision from other organisations. The level of proof required ranged from original DNACPR form acceptable only (n=1) to photocopies with ink signature / legible signature accepted (n=2) and form not necessarily needed to be seen (n=1). Six policies did not specify the requirements on the level of proof of a valid DNACPR decision. Acceptable formats of DNACPR decisions varied considerably. Two Trusts recognised that DNACPR decisions come in a variety of formats and were willing to accept verbal and written DNACPR decisions (including letters, entry in patient notes and pro-forma). Another two Trusts accepted any kind of written decisions, while one Trust stated that a decision had to be presented on a DNACPR form. Three more Trusts required specific forms only. 7/10 policies specified details that staff need to check to establish the validity of the document. These included the patient's details, the review date, a list of items if the decision is not on a pro-forma, and the completeness of transport specific sections on the DNACPR form.



For peer review only - http://bmjopen.bmj.com/site/about/guidelines.xhtml

The portability of DNACPR decisions between organisations and health care settings (community / acute care) was one of the greatest sources of inconsistency and variation with limited guidance arriving from the national guidelines. These stress that 'any decisions about CPR should be communicated between healthcare professionals whenever a patient is transferred between establishments, ..., or is discharged. ...procedures must be in place to notify [the receiving organisation] of the patient's CPR status, and provide them with the necessary documentation.[3](p19) DNACPR decisions were portable in 13/26 acute Trusts and 8/12 community Trusts. An additional six acute Trusts' DNACPR decisions extended to include ambulance transport. The remaining acute Trusts (n=7) but only one community Trust had a system in place through which non-portable DNACPR decisions could be communicated between providers. The level of portability of DNACPR decisions in the remaining three community Trusts was unclear. The detail with which handover systems were described in the policies was generally greater in the community Trusts than the acute Trusts. An example of a clear, un-ambiguous pathway, describing transfer between and within acute and community care settings of a fully portable DNACPR form is provided in Box 1.

## Discussion

Our review of local DNACPR policies revealed that while some isolated aspects of the national guidelines were implemented consistently into local policy, there was generally huge variation between local policies in all areas of documentation, ethical and legal issues, the decision makers but first and foremost in the practical issues of DNACPR decision making, i.e. the validity, review and portability of decisions which greatly affect the interface between services. Some of the variation might be explained in part by the language used in the national guidelines leaving too much scope for interpretation and some might be justifiable by the need to adapt to local circumstances. Others reveal a lack of compliance with clear recommendations in the guidelines or a lack of compliance with national legislation such as the requirement to consider capacity when making DNACPR decisions. This is in contrast with the national guidelines which focus on ethical and legal issues related



For peer review only - http://bmjopen.bmj.com/site/about/guidelines.xhtml

to DNACPR decision making and include less guidance on the more practical issues of DNACPR decisions. The national guidelines make no reference to the validity and do not provide guidance on the frequency of review of DNACPR decisions. There is also limited guidance on the portability of decisions, i.e. whether decisions should be accepted automatically by health care providers outside the establishment of the primary decision maker and how the handover systems should be organised. The resulting variation in portability reflects the range of different systems that are in place across England of how DNACPR decisions are handled in English health care trusts.

While the abovementioned systems of handling DNACPR decisions confirm the variation in local policies of the different health care services there is also evidence of efforts at standardisation that are initiated by regional working groups rather than national leaders. Several regions in England have formulated unified policies with regional DNACPR forms to improve communication and handover across healthcare providers within one area. These initiatives are often sponsored by the relevant clinical commissioning groups and allow a consistent approach, easy recognition of forms and allow the decisions to cross the borders between primary and secondary healthcare. However, there is also evidence that signing up to regional policies and switching to unified forms is slow and challenging. Research is needed to understand the barriers that hinder Trusts to move from local to regional policies and forms as these barriers might also hamper the implementation of national guidelines or a possible future national policy.

The development of a national template or standardised policy to support clinicians and patients in decision making seems intuitively attractive. This is reflected in the Tracey Case, a case between the Tracey family and the Cambridge University Hospital NHS Foundation Trust over a DNACPR decision. This case was advanced against the Secretary of State accusing him of failing to publish national guidance for clear DNACPR decision making processes. However, in June 2014 the Court of Appeal rejected a legal necessity for a national policy.[5] A standardised policy might improve consistency in approach and reduce policy variation. It would reduce the need for doctors moving between Trusts to learn new policies each time they go to a new Trust. Furthermore, it would allow the development of some generic learning materials on DNACPR decision making for clinicians and patients.  However, the



For peer review only - http://bmjopen.bmj.com/site/about/guidelines.xhtml

poor implementation of national guidelines and the slow and patchy recognition of regional policies might suggest that there are barriers to standardisation that need to be understood and overcome. Furthermore, there is a need to research and understand issues in the implementation and translation of local policy into local clinical practice as this would not be addressed by a standardised policy.

It is questionable that a national policy on the same lines as the national guidelines would achieve consistency in practice unless the practical issues of validity, review and portability are addressed. Local policies need more guidance on the practical aspects to standardise approaches. This would hopefully have an impact on communication and handover of DNACPR decisions between healthcare providers. However, without evidence on which system works best it would be difficult to choose one system and subsequently mandate it for all health care Trusts. Another approach would be to support standardisation efforts and help unified policies to spread and award regional initiatives.

While it has been suggested that incorporating DNACPR forms into an overall treatment option form (Universal Form of Treatment Options - UFTO)[6] or into treatment escalation plans (TEP)[7] is associated with reduced patient harm[6] and improved communication with patients and relatives,[6 7] it is unlikely that an intervention that supports the initial decision making process will eliminate the problems of handover and communication between healthcare providers involved in the care of the patient.

Communication of DNACPR is a major issue. The guidelines covered the appropriateness of talking to patients or relevant others in detail, however, little guidance to aid implementation into local policies was provided. Most policies described DNACPR decision being made by clinicians in discussion with patients / relatives.  Future research should explore if shared decision-making leads to better satisfaction and outcomes.

*National and international context*

The current national and international literature illustrates that challenges around guidance for DNACPR decision making is evidenced at all three levels, i.e. national policy or guidelines, local policy and local practice.



For peer review only - http://bmjopen.bmj.com/site/about/guidelines.xhtml

While our research revealed that in England national guidelines are implemented inconsistently into local policy, a recent survey of UK DNACPR forms suggested that changes in the national guidelines were the main driver for DNACPR form amendments as it was the most frequent response.[8] However, the majority of responses included a variety of other drivers for change (55/71) which seems to confirm that the influence of the guidelines is variable across England. More concerning is that in a survey of specialist registrars over one quarter of respondents was unable to recommend a document or guideline for DNACPR decision making.[9] The survey identified views of specialist registrars that there were no current guidelines that cover the complexities and difficulties of DNACPR decisions and that the available guidelines contradicted themselves. This also seems to support our finding that national guidelines should be improved to provide clear recommendations in all aspects of DNACPR decision making.

Furthermore, national and international literature indicates that there are deficiencies in staff knowledge of local DNACPR policies[10] and relevant laws and regulations[11] which might be suggestive of a gap between policy and practice. For instance while our policy review suggested good compliance with national guidelines in terms of clarifying that the DNACPR decision related only to the act of resuscitation and did not apply to other aspects of care, Smith et al. (2006)[10] reported that about 15% of nurses and midwifes believed that antibiotics, physiotherapy and nasogastric feeding would be inappropriate for DNACPR patients. Additional knowledge gaps included uncertainties about which professional grade can make decisions; and health care professionals generally did not believe that informing the multidisciplinary team of DNACPR decisions was important.[10]

In contrast, facilitators for DNACPR decision making in the literature appear to focus on local practice. Imhof et al. (2011)[12] found that interdisciplinary decisions were important to avoid conflict and non-compliance. Their research stressed that integrating nurses' views and observations into the decision would lead to more successful outcomes in compliance with resuscitation decisions. Furthermore, they recommend that decision making should not be left to junior doctors because of the professional, human and relational expertise that is required.[12] However, involvement of nurses in the decision making process would require additional education of nurses to fill knowledge gaps in resuscitation legislation.[11]



For peer review only - http://bmjopen.bmj.com/site/about/guidelines.xhtml

Further facilitators to ease DNACPR decision making have been suggested. Myint et al. (2006)[9] suggested raising patient and public awareness of resuscitation and decisions around resuscitation. This is of interest as a review of articles in the lay-press revealed that newspapers in the UK portray an overoptimistic survival rate following cardiac arrest in the out-of hospital and in-hospital setting.[13] Further claims to improve DNACPR decision making were the implementation of a standard process in trust policies to record patients' wishes at admission[9] while Kim et al. (2007)[14] called for a systematic standardisation of DNACPR decisions in terms of standardised forms to improve end-of life decisions in the early stage of terminal cancer patients. A recent systematic review confirmed these findings by ranking standardisation of DNACPR forms and structured changes to the decision making process for instance at the time of admission as the two most promising interventions identified in the literature that could improve DNACPR decision making.[15] Aspects for the successful implementation of a standardised approach to DNACPR decision making were reported in the pre-hospital setting in two states in the USA.[16] It showed that the introduction of a standardised form together with a core protocol at the same time as in-service training proved valuable for its success.[16] Approach of the Emergency Medical Services boards to gain approval also helped protect from the development of multiple forms as well as using unambiguous language and ensuring immunity from litigation. Finally, education of the public through news articles about the service was thought to be a particular strength of the programme.

In terms of policy and guidance published research is less specific in their recommendations. A survey of 298 Irish consultant physicians found that only 21% were aware of a formal resuscitation policy in their hospital which is why the authors called for a national policy for resuscitation decision making to facilitate more widespread formulation of local policies.[17]

Overall, the national and international literature tends to address issues around the initial decision making process including the involvement of nurses, standardisation and lack of awareness of legislation. However, there appears to be a lack of research addressing issues relating to the interface of healthcare settings for safe handover of DNACPR decisions.



For peer review only - http://bmjopen.bmj.com/site/about/guidelines.xhtml

For peer review only

*Strengths and limitations*

While this research reviewed only a sample of local policies, these covered all areas of England and met our objectives as this sample sufficiently identified huge variation and a worrying number of different systems of how Trusts deal with DNACPR decisions across England. Furthermore, the review included policies from different types of Trusts which was important as these concern health care providers with different responsibilities. The ambulance services for instance are not primary decision makers of lasting DNACPR decisions and are therefore more concerned with the handover and checking the validity of decisions. This relates to lasting DNACPR decisions only. AS staff attending patients in cardiac arrest still have to make immediate but informed decisions on whether to commence CPR or when to stop CPR. However, this research concentrated on lasting DNACPR decisions that AS face when accepting a DNACPR patient from acute Trusts or the community.

Community Trusts are an assemblage of diverse health care providers. Their concern, therefore, concentrates on the handover, communication and consistent documentation of decisions within the Trust. This might explain why community services tended to describe handover systems in more detail than acute Trusts and why the proportion of portable decision and unified policies was greater among community than acute Trusts.

One potential limitation of our research is that only about 30% of the extracted data was checked by a second reviewer. This would be of concern if it applied to the subjective questions covering the questions around the validity and portability of DNACPR decisions. However, following good agreement between the two reviewers on early data extractions, the decision was made to limit the second review to the more subjective questions but include all policies in this focused second review.

As our research only addressed the implementation of national guidelines into local policies it does not highlight shortfalls in the implementation of national and local guidance into local practice. There is a need to investigate issues and possible solutions to improve uptake of guidance into local practice.

**Conclusions**



For peer review only - http://bmjopen.bmj.com/site/about/guidelines.xhtml

This review identified significant variation in English NHS Trusts approaches to DNACPR decision making. Gaps identified included practical guidance around when and how to communicate DNACPR decisions and in relation to the portability / transferability of decisions between healthcare settings. There is a need for greater consistency in Trusts approaches to DNACPR decision making.

### Contributor statement

GDP conceived the study and obtained funding. KF developed the detailed protocol and led the primary data extraction with help from RF. KF drafted the paper with GDP and RF revising it critically for important intellectual content. All authors approved the final version.

### Competing interests

GDP is a volunteer member of the Resuscitation Council (UK). The Resuscitation Council UK co-authored the joint national statement on DNACPR decisions.


### Funding

This project was funded by the National Institute for Health Research [Health Service Research and Delivery Programme, project number 12/5001/55]. The views and opinions expressed therein are those of the authors and do not necessarily reflect those of the [Health Service Research and Delivery Programme], NIHR, NHS or the Department of Health.


GDP is a Director of Research for the Intensive Care Foundation.

### Data sharing

There are no additional data available.



For peer review only - http://bmjopen.bmj.com/site/about/guidelines.xhtml

## References

1. Great Britain. Human Rights Act 1998. [online]. http://www.legislation.gov.uk/ukpga/1998/42/contents (accessed 16 June 2014).
2. Department for Constitutional Affairs. Mental capacity Act 2005: Code of practice. 2007. https://www.justice.gov.uk/downloads/protecting-the-vulnerable/mca/mca-code-practice-0509.pdf (accessed 22 May 2014).
3. British Medical Association, Resuscitation Council (UK), and Royal College of Nursing. Decisions relating to cardiopulmonary resuscitation: A joint statement from the British Medical Association, the Resuscitation Council (UK) and the Royal College of Nursing 2007. http://www.resus.org.uk/pages/dnar.pdf (accessed 16 June 2014).
4. National Health Service. NHS Choices. Service Directories Authorities and Trusts. http://www.nhs.uk/ServiceDirectories/Pages/AcuteTrustListing.aspx (accessed 16 June 2014).
5. In the Court of Appeal (Civil Division) Case No: C1/2013/0045. 2014. http://www.capsticks.com/assets/Uploads/Marketing-materials/Articles/Tracey-v-Cambridge-University-Hospital-NHS-Foundation-Trust-and-Others-judgement.pdf (accessed 24 Sept 2014).
6. Fritz Z, Malyon A, Frankau JM, et al. The Universal Form of Treatment Options (UFTO) as an alternative to Do Not Attempt Cardiopulmonary Resuscitation (DNACPR) orders: a mixed methods evaluation of the effects on clinical practice and patient care. PloS one 2013;**8**(9):e70977.
7. Obolensky L, Clark T, Matthew G, et al. A patient and relative centred evaluation of treatment escalation plans: a replacement for the do-not-resuscitate process. Journal of medical ethics 2010;**36**(9):518-20.
8. Clements M, Fuld J, Fritz Z. Documentation of resuscitation decision-making: a survey of practice in the United Kingdom. Resuscitation 2014;**85**(5):606-11.
9. Myint PK, Miles S, Halliday DA, et al. Experiences and views of specialist registrars in geriatric medicine on 'do not attempt resuscitation' decisions: a sea of uncertainty? QJM : monthly journal of the Association of Physicians 2006;**99**(10):691-700.
10. Smith GB, Poplett N, Williams D. Staff awareness of a 'Do Not Attempt Resuscitation' policy in a District General Hospital. Resuscitation 2005;**65**(2):159-63.
11. Saevareid TJ, Balandin S. Nurses' perceptions of attempting cardiopulmonary resuscitation on oldest old patients. Journal of advanced nursing 2011;**67**(8):1739-48.
12. Imhof L, Mahrer-Imhof R, Janisch C, et al. Do not attempt resuscitation: the importance of consensual decisions. Swiss medical weekly 2011;**141**:w13157.
13. Field RA, Soar J, Nolan JP, et al. Epidemiology and outcome of cardiac arrests reported in the lay-press: an observational study. Journal of the Royal Society of Medicine 2011;**104**(12):525-31.
14. Kim do Y, Lee KE, Nam EM, et al. Do-not-resuscitate orders for terminal patients with cancer in teaching hospitals of Korea. Journal of palliative medicine 2007;**10**(5):1153-8.
15. Field RA, Fritz Z, Baker A, et al. Systematic Review of Interventions to Improve Appropriate Use and Outcomes Associated with Do-Not-Attempt-



For peer review only - http://bmjopen.bmj.com/site/about/guidelines.xhtml

Cardiopulmonary-Resuscitation Decisions. Resuscitation 2014 doi: 10.1016/j.resuscitation.2014.08.024. [Epub ahead of print]
16. Sosna DP, Christopher M, Pesto MM, et al. Implementation strategies for a do-not-resuscitate program in the prehospital setting. Annals of emergency medicine 1994;**23**(5):1042-6.
17. Butler MW, Saaidin N, Sheikh AA, et al. Dissatisfaction with Do Not Attempt Resuscitation Orders: A nationwide study of Irish consultant physician practices. Irish medical journal 2006;**99**(7):208-10.

For peer review only

**Figures and tables**

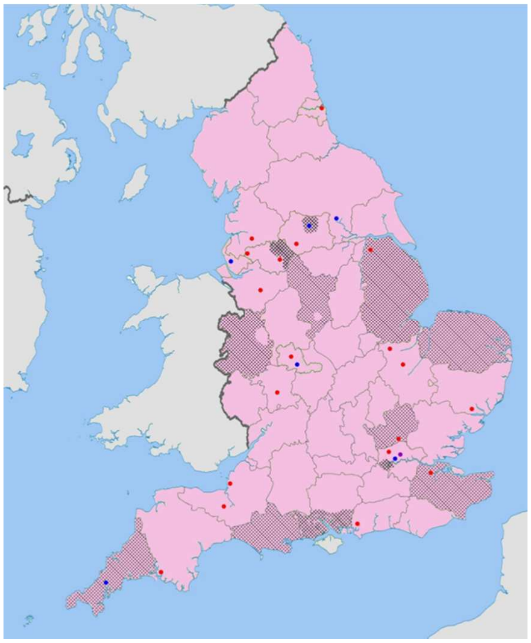

**Figure 1** Overview and geographic coverage of local Trust DNACPR policies included in the review
(•Teaching hospital; •District general hospital; • Specialist hospital; ▩ Community healthcare Trust)



For peer review only - http://bmjopen.bmj.com/site/about/guidelines.xhtml

**Table 1** Overview of variation in type of local DNACPR policies by Trust type

| **Policy type item** | **Acute Trusts (n=26), n (%)** | **Community Trusts (n=12), n (%)** | **AS Trusts (n=10), n (%)** | **Total, N=48, n (%)** |
|---|---|---|---|---|
| TYPE OF DOCUMENT | | | | |
| Policy | 22 (85%) | 12 (100%) | 7 (70%) | 41 (85%) |
| Procedure | 2 (8%) | 0 | 1 (10%) | 3 (6%) |
| Guideline | 2 (8%) | 0 | 2 (20%) | 4 (8%) |
| TERMINOLOGY | | | | |
| DNAR | 6 (23%) | 3 (25%) | 3 (30%) | 12 (25%) |
| DNACPR | 16 (62%) | 8 (67%) | 7 (70%) | 31 (65%) |
| Not for CPR | 3 (12%) | 0 | 0 | 3 (6%) |
| AND | 1 (4%) | 2 (17%)* | 0 | 3 (6%) |
| VISIBILITY | | | | |
| Stand alone | 21 (81%) | 9 (75%) | 3 (30%) | 33 (69%) |
| Integrated into resuscitation policy | 5 (19%) | 3 (25%) | 3 (30%) | 11 (23%) |
| Mentioned in other policies | 0 | 0 | 4 (40%) | 4 (8%) |
| COVERAGE | | | | |
| Local | 23 (88%) | 9 (75%) | 8 (80%) | 40 (83%) |
| Regional | 3 (12%) | 3 (25%) | 2 (20%) | 8 (17%) |

*Column does not add up to 12 because one Trusts consciously and consistently referred to DNACPR/AND throughout the policy

(Abbreviations: AS Ambulance service, DNAR do not attempt resuscitation, DNACPR do not attempt cardiopulmonary resuscitation, CPR cardiopulmonary resuscitation, AND allow natural death)

For peer review only



For peer review only - http://bmjopen.bmj.com/site/about/guidelines.xhtml

**Table 2** Overview of adoption of RC (UK) DNACPR forms and type of form by acute, community and AS Trusts

| DNACPR form | Acute Trusts (n=26), n(%) | Community Trusts (n=12), n (%) | AS Trusts (n=10), n (%) | Total, N=48, n (%) |
|---|---|---|---|---|
| RC (UK) | 2 (7.8%) | 3 (25%) | 0 | 5 (10.4%) |
| Modified | 4 (15.4%) | 3 (25%) | 1 (10%) | 8 (16.7%) |
| Bespoke | 16 (61.5%) | 4 (33.3%) | 4 (40%) | 24 (50%) |
| Form not sent | 4 (15.4%) | 2 (16.6%) | 5 (50%) | 11 (22.9%) |
| Type of form* | | | | |
| Paper | 24 (92.3%) | 10 (83.3%) | 5 (50%) | 39 (81.3%) |
| Electronic | 1 (3.8%) | 0 | 0 | 1 (2.1%) |
| Both | 1 (3.8%) | 2 (16.6%) | 0 | 3 (6.3%) |

*Insufficient information was available in policies from 5 Trusts for this question
(Abbreviations: RC (UK) Resuscitation Council (UK), DNACPR do not attempt cardiopulmonary resuscitation, AS ambulance service)

For peer review only

**Box 1** Example of clear pathway for handover of DNACPR decision from one acute trust

Review the appropriateness of the DNACPR decision before discharge

If on review the DNACPR is still considered appropriate carry decision over to the patient's care setting/home on discharge including the following considerations:

- Liaise with the patient's GP and identify an agreed, appropriate community review date (ideally within 24 hours), which should be documented on the DNACPR form
- Send the original DNACPR form (top white copy) with a review date in place with the patient
- Communicate and discuss sensitively with the patient
- Leave the yellow copy (second copy) in the front of the patient's medical hospital notes facilitating early consideration of resuscitation issues on any potential subsequent readmission

Communication with Ambulance Service and / or ongoing care setting must take place

Before Ambulance transfer complete appropriate section on the DNACPR form

When booking transport for DNACPR patient from acute Trust fax DNACPR form to ambulance control Contact Centre

Within community settings, hand DNACPR forms directly to the attending crew on arrival or fax to AS Contact Centre

The DNACPR status must also be recorded by the attending AS crew on the AS Patient Report Form

For peer review only



For peer review only - http://bmjopen.bmj.com/site/about/guidelines.xhtml

# Variation in local Trust Do Not Attempt Cardiopulmonary Resuscitation policies: a review of 48 English health care trusts

Karoline Freeman[1], Richard A. Field[1,2], Gavin D. Perkins[1,2]

[1] Warwick Medical School, University of Warwick, Coventry, CV4 7AL

[2] Heart of England NHS Foundation Trust, Bordesley Green East, Birmingham, B9 5SS

Corresponding author:

## Abstract

**Background:** National guidelines for do not attempt cardiopulmonary resuscitation (DNACPR) decisions exist, but implementation is dependent on local policy. This study examined DNACPR policies from English acute, community and ambulance service Trusts to explore consistencies and variation in implementation of national guidelines between healthcare organisations.

**Methods:** Freedom of information requests for adult DNACPR policies were sent to a random sample of 48 English health care Trusts. DNACPR policies were assessed on aspects identified from national guidelines including documentation, ethical and legal issues, decision makers and involvement of others in DNACPR decisions as well as practical considerations such as validity, review and portability of decisions.

**Results:** Policies from 26 acute, 12 community and 10 ambulance service Trusts were reviewed. There was variation in terminology used (85% described documents as policies, 6% procedures and 8% guidelines). Only one quarter of Trusts used the recommended Resuscitation Council (UK) record form (or a modification of the form). There was variation in the terminology used which included DNAR, DNACPR, Not for CPR and AND (allow natural death). Accountability for DNACPR decisions rested with consultants at all acute Trusts and the most senior clinician at community Trusts. Most Trusts (74%) recommended discussion of decisions with a multi-disciplinary team. Compliance with guidance requiring clinical staff to assess the



For peer review only - http://bmjopen.bmj.com/site/about/guidelines.xhtml

patient for capacity and when to consult a lasting power of attorney or independent mental capacity advocate occurred less commonly. There was wide variation in the duration of time over which a DNACPR decision was considered valid as well as in the Trusts' approach to reviewing DNACPR decisions. The level of portability of DNACPR decisions between health care organisations was one of the greatest sources of variation.

**Conclusions:** There is significant variation in the translation of the national DNACPR guidelines into English health care Trusts' DNACPR policies.

**Keywords: Do not attempt cardiopulmonary resuscitation, DNACPR, policy, national guideline, review**

**Strengths and limitations of this study**

- The study evaluated a sample of DNACPR policies and procedures from Acute, Community and Ambulance Service Trusts in England
- The study identified marked variation in NHS organisations' approaches to DNACPR policy and practice
- Key areas for strengthening current approaches were identified and included improving consistency for recording decisions and ensuring decisions are transferable between healthcare settings
- Whether improving consistency in DNACPR policies will translate to improved implementation into practice and patient experience requires further study
- The findings require confirmation in other settings



For peer review only - http://bmjopen.bmj.com/site/about/guidelines.xhtml

## Background

Decisions relating to Do Not Attempt Cardiopulmonary Resuscitation (DNACPR) are complex and require tried and tested decision making processes as well as handover systems involving the patient, relevant others and a multidisciplinary healthcare team. Initiation of discussions of DNACPR is likely to occur if there is clinical evidence that CPR would be futile, that resulting harm would outweigh potential benefits or if a patient refuses CPR treatment. DNACPR decisions do not involve decisions about any other acute, life-saving treatments. In England the Human Rights Act 1998[1] and the Mental Capacity Act 2005[2] provide the legal basis for DNACPR decision making. The Human Rights Act 1998[1] covers fundamental rights such as the right to life, the right to be free from inhuman and degrading treatment and the right to hold opinions and to receive information. The Mental Capacity Act 2005[2] works under the assumption that every adult is able to make their own decisions unless a mental capacity assessment shows otherwise. The latter sets out how to carry out an assessment of capacity, describes who can make decisions for people who lack capacity and provides a checklist to ensure that any decision taken on behalf of a person without capacity is in their best interest. Based on these laws, national guidelines[3] in England describe the context, setting and process for making informed decisions to omit CPR and provide a framework to support decisions relating to CPR. Nonetheless, recent high profile cases in the media suggest that issues exist in terms of DNACPR decision making in English health care settings claiming lack of consistency in approach across England. It is, therefore, of interest to understand the impact that the national guidelines have on local policies which, in the absence of a national DNACPR policy, determine local practice. By taking the view that current guidelines are informed primarily by ethical and legal considerations with little focus on the available research evidence there might be room for improvement in the practical guidance available. Furthermore, the guidelines provide general principles that require tailoring to local circumstances, which suggests that there may be room for interpretation of national guidelines when implemented into local policy. We therefore reviewed a random sample of local DNACPR policies from acute, community and ambulance services Trusts across England and mapped them against aspects from national guidelines to identify variation and consistencies between and within Trust types and inconsistencies in implementation of national guidelines.



For peer review only - http://bmjopen.bmj.com/site/about/guidelines.xhtml

## Methods

Using the NHS service directory,[4] we obtained NHS care Trust lists of acute, community and AS service Trust. We identified a random sample of 20 acute hospital Trusts for review: Trust lists were numbered and, using a random number generator in EXCEL (using the RAND and INDEX functions), a random list without duplication was created. The first 20 trusts on this list were included in the review. After mapping a further six Trusts were chosen to ensure geographical coverage. All 10 ambulance service (AS) Trusts and a random sample of 12 community health Trusts were further included using the same method as described for acute Trusts. Freedom of information requests for adult DNACPR policies were sent to the sample of English acute Trusts, ambulance service Trusts and community Trusts. If a separate DNACPR policy was not available the Trust's resuscitation policy was requested. Follow-up e-mails were sent once if (1) the wrong policy was sent, (2) additional information was apparently available on reading of the policy or (3) the health Trust had not responded after two months.

Additional information given in the accompanying e-mails by the Trust on further policies and validity of the policy was considered in the data extraction process as was information on the back of the DNACPR form if provided.

A data extraction form was established using aspects ~~important for DNACPR decision making~~ from the joint statement by the Resuscitation Council (RC (UK)), the British Medical Association (BMA) and the Royal College of Nursing (RCN) on decisions relating to cardiopulmonary resuscitation.[3] Aspects were chosen that were deemed important for DNACPR decision making by the expert advisory group including national leads in DNACPR guidelines, practicing clinicians in acute, community and palliative care as well as patient representatives. The resulting extraction form was piloted by two reviewers and was subsequently adjusted. The final form included 26 questions on aspects taken from the national guidelines including documentation, ethical and legal issues, DNACPR decision makers and involvement of others in DNACPR decisions as well as practical considerations such as validity, review period and portability of decisions (see electronic supplement for data extraction table E1). Policies were then read in their entirety by one researcher



For peer review only - http://bmjopen.bmj.com/site/about/guidelines.xhtml

(KF) and data extracted using the agreed form. Queries during the data extraction process were discussed and agreed with GDP. 30% of data extractions were checked by a second reviewer (RF) and confirmed as accurate, therefore further checking was not undertaken.

Data were synthesised quantitatively ~~(documentation of DNACPR decisions, implementation of legal and ethical aspects, decision makers and involvement of others)~~ and narratively ~~(practical considerations)~~. Quantitative synthesis involved the reporting of frequencies of responses per question in tables for the paragraphs on documentation of DNCAPR decisions, implementation of legal and ethical aspects, decision makers and involvement of others. These covered the questions that involved answers chosen from a list of limited options (e.g. yes / no / uncertain). For the questions concerning the validity, review and portability of decisions the review of policies identified substantial variation in local policy documents. Comprehensive data extraction was undertaken to capture all identified variation in the policies in these three areas in separate documents using descriptive coding. The main categories of the topic areas were then identified by discussion through clustering of the codes between the three authors. The categories were presented to the project and advisory groups for consensus and subsequently formed the basis for possible responses to the questions on the data extraction form. In order to retain the breadth and depth of the variation within the categories the data presented in the paragraph 'practical considerations' was synthesised narratively. The major areas of interest included the validity, review and portability including handover of decisions.

**Results**

A total of 48 local DNACPR policies were reviewed (100% response rate). The 26 acute Trusts consisted of six teaching hospitals, 19 district general hospitals and one specialist centre.  Good geographical coverage of England was achieved with the reviewed policies from 26/156 acute and 12/24 community Trusts ~~(N=12)~~ (see Figure 1). All ten English AS were considered.

*Documentation*



For peer review only - http://bmjopen.bmj.com/site/about/guidelines.xhtml

The Trusts produced specific local documents referred to as policies (85%), procedures (6%) or guidelines (8%) (Table 1).There was variation in the terms used to describe resuscitation decisions. While the national guidelines recommend the use of the phrase ‘do not attempt cardiopulmonary resuscitation’ to avoid confusion,[3] two thirds of Trusts referred to the term “DNACPR”, a quarter to “DNAR” and the remainders to “Not for CPR” or “Allow Natural Death”. Half the Trusts had reviewed the relevant policy/guideline/procedure (referred to from here under the uniform term “policy”) within the preceding 12 months (range 1-47 months). One quarter of policies were outside the review period set by the Trust while half indicated the expired policy was currently under active review. Three quarters of policies reported having undergone an equality and diversity assessment.

There was variation in how DNACPR decisions were recorded (Table 2). The guidelines clearly state that there is some benefit in using a standardised form and recommend the adoption of the RC(UK) model form.[3] In contrast, only one quarter of Trusts used the RC (UK) DNACPR form or a modified version. As a general guide, modifications included adding or changing a couple of questions but required the general layout to be the same as the RC (UK) form while any more substantial changes would make the form bespoke. The majority of forms were paper forms (81%) with only 8% of Trusts using electronic versions. Health care professionals making the DNACPR decision were obligated to sign the form, which also had room for a countersignature by the consultant if a junior doctor initiated the decision which needs endorsement. In one specific case the form required that the doctor’s signature must be witnessed by the registered nurse who was involved in the discussion on the DNACPR decision.

*Ethical and legal basis for DNACPR decisions*

The joint statement “Decisions relating to Cardiopulmonary Resuscitation” from the British Medical Association, the Resuscitation Council (UK)and the Royal College of Nursing was the most frequently cited source of national guidance for acute and community NHS Trusts. Reference to relevant legislation (Mental Capacity Act 89%, Human Rights Act, 78%) was common. General Medical Council guidelines were



For peer review only - http://bmjopen.bmj.com/site/about/guidelines.xhtml

highlighted less frequently (50%). 9/10 Ambulance Trusts additionally cited the Joint Royal College Ambulance Liaison Committee.

The national guidance that DNACPR decisions may be made on the basis of futility, overall benefit or patient refusal was incorporated into all acute and community Trust policies. Most policies clarified that the DNACPR decision related only to the act of resuscitation and did not apply to other aspects of care.

*Decision makers and involvement of others in DNACPR decisions*

All acute and community Trusts were responsible for primary DNACPR decisions whilst ambulance Trusts were not primary decision makers of lasting DNACPR decisions. The review identified variation in the grade of clinical staff authorised to make an initial DNACPR decision. Authority was delegated to senior nursing staff at 7 Trusts (1 limited to MacMillan Nurses, 3 for community decisions only, 3 appropriately qualified nurses) or junior medical staff (foundation year doctors (n=3), specialist trainee doctors (n=15), most senior available / any grade (n=6) or undefined / unclear (n=3)). Accountability for DNACPR decisions rested with consultants at all acute Trusts and the most senior clinician who may be a GP, consultant or nurse depending on circumstances at community Trusts. This is in line with the national guidelines which state that local policy should define who the most senior person in charge of the DNACPR decision is. However, the guidelines do not make any recommendations on the staff grade that can initiate decisions.[3] Few (8%) of the Trusts mandated medical staff to discuss decisions with others within the multidisciplinary team although most (74%) recommended discussion, a fifth made no such recommendations.

Acute Trusts recommended staff to talk to patients and relatives in 100% of reviewed policies and community Trusts in 100% to patients and 92% to relatives. Guidance requiring clinical staff to assess the patient for capacity and guidance about when to consult a lasting power of attorney or independent mental capacity advocate occurred less commonly. Fifty percent of Trusts recommended the use of a patient information leaflet. Less than one in ten Trusts provided practical guidance on how



For peer review only - http://bmjopen.bmj.com/site/about/guidelines.xhtml

to approach DNACPR decision making in different cultures. (See electronic supplement Tables E2 to E4 for more information on these aspects.)

*Practical issues of DNACPR decision making: Validity, review and portability of DNACPR decisions*

The national guidelines provide no guidance on how long DNACPR decisions should be valid and make no reference to the validity of decisions other than that the validity of decisions needs to be confirmed by receiving health care providers before accepting formal DNACPR decisions.[3] The duration of time over which a DNACPR decision was considered valid varied widely across local policies. DNACPR decisions from acute Trusts ranged from valid indefinitely (54%) to valid for the duration of one hospital admission only (31%) and valid till a specific point in time (8%) or up until a specified review date (4%). Similarly, in community trusts validity ranged from valid indefinitely (42%) to valid up until a specified review date (42%). Two community Trusts had no information in the policies about the validity of decisions and the duration of validity in one acute Trust was unclear. There was similarly wide variation in Trusts' approach to reviewing DNACPR decisions with timings ranging from 24 hours to months. (See electronic supplement for detailed information.) In terms of review the guidelines state that decisions must be reviewed regularly and that the frequency of review should be determined by the health professional in charge.[3]

Ambulance services focused on the requirements to recognise a valid DNACPR decision from other organisations. The level of proof required ranged from original DNACPR form acceptable only (n=1) to photocopies with ink signature / legible signature accepted (n=2) and form not necessarily needed to be seen (n=1). Six policies did not specify the requirements on the level of proof of a valid DNACPR decision. Acceptable formats of DNACPR decisions varied considerably. Two Trusts recognised that DNACPR decisions come in a variety of formats and were willing to accept verbal and written DNACPR decisions (including letters, entry in patient notes and pro-forma). Another two Trusts accepted any kind of written decisions, while one Trust stated that a decision had to be presented on a DNACPR form. Three more Trusts required specific forms only. 7/10 policies specified details that staff need to



For peer review only - http://bmjopen.bmj.com/site/about/guidelines.xhtml

check to establish the validity of the document. These included the patient's details, the review date, a list of items if the decision is not on a pro-forma, and the completeness of transport specific sections on the DNACPR form.

The portability of DNACPR decisions between organisations and health care settings (community / acute care) was one of the greatest sources of inconsistency and variation with limited guidance arriving from the national guidelines. These stress that 'any decisions about CPR should be communicated between healthcare professionals whenever a patient is transferred between establishments, ..., or is discharged. ...procedures must be in place to notify [the receiving organisation] of the patient's CPR status, and provide them with the necessary documentation.[3](p19) DNACPR decisions were portable in 13/26 acute Trusts and 8/12 community Trusts. An additional six acute Trusts' DNACPR decisions extended to include ambulance transport. The remaining acute Trusts (n=7) but only one community Trust had a system in place through which non-portable DNACPR decisions could be communicated between providers. The level of portability of DNACPR decisions in the remaining three community Trusts was unclear. The detail with which handover systems were described in the policies was generally greater in the community Trusts than the acute Trusts. An example of a clear, un-ambiguous pathway, describing transfer between and within acute and community care settings of a fully portable DNACPR form is provided in Box 1.

## Discussion

Our review of local DNACPR policies revealed that while some isolated aspects of the national guidelines were implemented consistently into local policy, there was generally huge variation between local policies in all areas of documentation, ethical and legal issues, the decision makers but first and foremost in the practical issues of DNACPR decision making, i.e. the validity, review and portability of decisions which greatly affect the interface between services. Some of the variation might be explained in part by the language used in the national guidelines leaving too much scope for interpretation and some might be justifiable by the need to adapt to local circumstances. Others reveal a lack of compliance with clear recommendations in



For peer review only - http://bmjopen.bmj.com/site/about/guidelines.xhtml

the guidelines or a lack of compliance with national legislation such as the requirement to consider capacity when making DNACPR decisions. This is in contrast with the national guidelines which focus on ethical and legal issues related to DNACPR decision making and include less guidance on the more practical issues of DNACPR decisions. The national guidelines make no reference to the validity and do not provide guidance on the frequency of review of DNACPR decisions. There is also limited guidance on the portability of decisions, i.e. whether decisions should be accepted automatically by health care providers outside the establishment of the primary decision maker and how the handover systems should be organised. The resulting variation in portability reflects the range of different systems that are in place across England of how DNACPR decisions are handled in English health care trusts.

While the abovementioned systems of handling DNACPR decisions confirm the variation in local policies of the different health care services there is also evidence of efforts at standardisation that are initiated by regional working groups rather than national leaders. Several regions in England have formulated unified policies with regional DNACPR forms to improve communication and handover across healthcare providers within one area. These initiatives are often sponsored by the relevant clinical commissioning groups and allow a consistent approach, easy recognition of forms and allow the decisions to cross the borders between primary and secondary healthcare. However, there is also evidence that signing up to regional policies and switching to unified forms is slow and challenging. Research is needed to understand the barriers that hinder Trusts to move from local to regional policies and forms as these barriers might also hamper the implementation of national guidelines or a possible future national policy.

The development of a national template or standardised policy to support clinicians and patients in decision making seems intuitively attractive. This is reflected in the Tracey Case, a case between the Tracey family and the Cambridge University Hospital NHS Foundation Trust over a DNACPR decision. This case was advanced against the Secretary of State accusing him of failing to publish national guidance for clear DNACPR decision making processes. However, in June 2014 the Court of Appeal rejected a legal necessity for a national policy.[5] A standardised policy might improve consistency in approach and reduce policy variation. It would reduce the



For peer review only - http://bmjopen.bmj.com/site/about/guidelines.xhtml

need for doctors moving between Trusts to learn new policies each time they go to a new Trust. Furthermore, it would allow the development of some generic learning materials on DNACPR decision making for clinicians and patients. However, the poor implementation of national guidelines and the slow and patchy recognition of regional policies might suggest that there are barriers to standardisation that need to be understood and overcome. Furthermore, there is a need to research and understand issues in the implementation and translation of local policy into local clinical practice as this would not be addressed by a standardised policy.

It is questionable that a national policy on the same lines as the national guidelines would achieve consistency in practice unless the practical issues of validity, review and portability are addressed. Local policies need more guidance on the practical aspects to standardise approaches. This would hopefully have an impact on communication and handover of DNACPR decisions between healthcare providers. However, without evidence on which system works best it would be difficult to choose one system and subsequently mandate it for all health care Trusts. Another approach would be to support standardisation efforts and help unified policies to spread and award regional initiatives.

While it has been suggested that incorporating DNACPR forms into an overall treatment option form (Universal Form of Treatment Options - UFTO)[6] or into treatment escalation plans (TEP)[7] is associated with reduced patient harm[6] and improved communication with patients and relatives,[6 7] it is unlikely that an intervention that supports the initial decision making process will eliminate the problems of handover and communication between healthcare providers involved in the care of the patient.

Communication of DNACPR is a major issue. The guidelines covered the appropriateness of talking to patients or relevant others in detail, however, little guidance to aid implementation into local policies was provided. Most policies described DNACPR decision being made by clinicians in discussion with patients / relatives. Future research should explore if shared decision-making leads to better satisfaction and outcomes.

*National and international context*



For peer review only - http://bmjopen.bmj.com/site/about/guidelines.xhtml

The current national and international literature illustrates that challenges around guidance for DNACPR decision making is evidenced at all three levels, i.e. national policy or guidelines, local policy and local practice.

While our research revealed that in England national guidelines are implemented inconsistently into local policy, a recent survey of UK DNACPR forms suggested that changes in the national guidelines were the main driver for DNACPR form amendments as it was the most frequent response.[8] However, the majority of responses included a variety of other drivers for change (55/71) which seems to confirm that the influence of the guidelines is variable across England. More concerning is that in a survey of specialist registrars over one quarter of respondents was unable to recommend a document or guideline for DNACPR decision making.[9] The survey identified views of specialist registrars that there were no current guidelines that cover the complexities and difficulties of DNACPR decisions and that the available guidelines contradicted themselves. This also seems to support our finding that national guidelines should be improved to provide clear recommendations in all aspects of DNACPR decision making.

Furthermore, national and international literature indicates that there are deficiencies in staff knowledge of local DNACPR policies[10] and relevant laws and regulations[11] which might be suggestive of a gap between policy and practice. For instance while our policy review suggested good compliance with national guidelines in terms of clarifying that the DNACPR decision related only to the act of resuscitation and did not apply to other aspects of care, Smith et al. (2006)[10] reported that about 15% of nurses and midwifes believed that antibiotics, physiotherapy and nasogastric feeding would be inappropriate for DNACPR patients. Additional knowledge gaps included uncertainties about which professional grade can make decisions; and health care professionals generally did not believe that informing the multidisciplinary team of DNACPR decisions was important.[10]

In contrast, facilitators for DNACPR decision making in the literature appear to focus on local practice. Imhof et al. (2011)[12] found that interdisciplinary decisions were important to avoid conflict and non-compliance. Their research stressed that integrating nurses' views and observations into the decision would lead to more successful outcomes in compliance with resuscitation decisions. Furthermore, they



For peer review only - http://bmjopen.bmj.com/site/about/guidelines.xhtml

recommend that decision making should not be left to junior doctors because of the professional, human and relational expertise that is required.[12] However, involvement of nurses in the decision making process would require additional education of nurses to fill knowledge gaps in resuscitation legislation.[11]

Further facilitators to ease DNACPR decision making have been suggested. Myint et al. (2006)[9] suggested raising patient and public awareness of resuscitation and decisions around resuscitation. This is of interest as a review of articles in the lay-press revealed that newspapers in the UK portray an overoptimistic survival rate following cardiac arrest in the out-of hospital and in-hospital setting.[13] Further claims to improve DNACPR decision making were the implementation of a standard process in trust policies to record patients' wishes at admission[9] while Kim et al. (2007)[14] called for a systematic standardisation of DNACPR decisions in terms of standardised forms to improve end-of life decisions in the early stage of terminal cancer patients. A recent systematic review confirmed these findings by ranking standardisation of DNACPR forms and structured changes to the decision making process for instance at the time of admission as the two most promising interventions identified in the literature that could improve DNACPR decision making.[15] Aspects for the successful implementation of a standardised approach to DNACPR decision making were reported in the pre-hospital setting in two states in the USA.[16] It showed that the introduction of a standardised form together with a core protocol at the same time as in-service training proved valuable for its success.[16] Approach of the Emergency Medical Services boards to gain approval also helped protect from the development of multiple forms as well as using unambiguous language and ensuring immunity from litigation. Finally, education of the public through news articles about the service was thought to be a particular strength of the programme.

In terms of policy and guidance published research is less specific in their recommendations. A survey of 298 Irish consultant physicians found that only 21% were aware of a formal resuscitation policy in their hospital which is why the authors called for a national policy for resuscitation decision making to facilitate more widespread formulation of local policies.[17]



For peer review only - http://bmjopen.bmj.com/site/about/guidelines.xhtml

Overall, the national and international literature tends to address issues around the initial decision making process including the involvement of nurses, standardisation and lack of awareness of legislation. However, there appears to be a lack of research addressing issues relating to the interface of healthcare settings for safe handover of DNACPR decisions.

*Strengths and limitations*

While this research reviewed only a sample of local policies, these covered all areas of England and met our objectives as this sample sufficiently identified huge variation and a worrying number of different systems of how Trusts deal with DNACPR decisions across England. Furthermore, the review included policies from different types of Trusts which was important as these concern health care providers with different responsibilities. The ambulance services for instance are not primary decision makers of lasting DNACPR decisions and are therefore more concerned with the handover and checking the validity of decisions. This relates to lasting DNACPR decisions only. AS staff attending patients in cardiac arrest still have to make immediate but informed decisions on whether to commence CPR or when to stop CPR. However, this research concentrated on lasting DNACPR decisions that AS face when accepting a DNACPR patient from acute Trusts or the community.

Community Trusts are an assemblage of diverse health care providers. Their concern, therefore, concentrates on the handover, communication and consistent documentation of decisions within the Trust. This might explain why community services tended to describe handover systems in more detail than acute Trusts and why the proportion of portable decision and unified policies was greater among community than acute Trusts.

One potential limitation of our research is that only about 30% of the extracted data was checked by a second reviewer. This would be of concern if it applied to the subjective questions covering the questions around the validity and portability of DNACPR decisions. However, following good agreement between the two reviewers on early data extractions, the decision was made to limit the second review to the more subjective questions but include all policies in this focused second review.



For peer review only - http://bmjopen.bmj.com/site/about/guidelines.xhtml

As our research only addressed the implementation of national guidelines into local policies it does not highlight shortfalls in the implementation of national and local guidance into local practice. There is a need to investigate issues and possible solutions to improve uptake of guidance into local practice.

## Conclusions

This review identified significant variation in English NHS Trusts approaches to DNACPR decision making. Gaps identified included practical guidance around when and how to communicate DNACPR decisions and in relation to the portability / transferability of decisions between healthcare settings. There is a need for greater consistency in Trusts approaches to DNACPR decision making.

## Acknowledgement

This project was funded by the National Institute for Health Research [Health Service Research and Delivery Programme, project number 12/5001/55]. The views and opinions expressed therein are those of the authors and do not necessarily reflect those of the [Health Service Research and Delivery Programme], NIHR, NHS or the Department of Health.

GDP is a Director of Research for the Intensive Care Foundation.

## References

1. Great Britain. Human Rights Act 1998. [online]. http://www.legislation.gov.uk/ukpga/1998/42/contents (accessed 16 June 2014).
2. Department for Constitutional Affairs. Mental capacity Act 2005: Code of practice. 2007. https://www.justice.gov.uk/downloads/protecting-the-vulnerable/mca/mca-code-practice-0509.pdf (accessed 22 May 2014).
3. British Medical Association, Resuscitation Council (UK), and Royal College of Nursing. Decisions relating to cardiopulmonary resuscitation: A joint statement from the British Medical Association, the Resuscitation Council (UK) and the Royal College of Nursing 2007. http://www.resus.org.uk/pages/dnar.pdf (accessed 16 June 2014).



For peer review only - http://bmjopen.bmj.com/site/about/guidelines.xhtml

4. National Health Service. NHS Choices. Service Directories Authorities and Trusts. http://www.nhs.uk/ServiceDirectories/Pages/AcuteTrustListing.aspx (accessed 16 June 2014).
5. In the Court of Appeal (Civil Division) Case No: C1/2013/0045. 2014. http://www.capsticks.com/assets/Uploads/Marketing-materials/Articles/Tracey-v-Cambridge-University-Hospital-NHS-Foundation-Trust-and-Others-judgement.pdf (accessed 24 Sept 2014).
6. Fritz Z, Malyon A, Frankau JM, et al. The Universal Form of Treatment Options (UFTO) as an alternative to Do Not Attempt Cardiopulmonary Resuscitation (DNACPR) orders: a mixed methods evaluation of the effects on clinical practice and patient care. PloS one 2013;**8**(9):e70977.
7. Obolensky L, Clark T, Matthew G, et al. A patient and relative centred evaluation of treatment escalation plans: a replacement for the do-not-resuscitate process. Journal of medical ethics 2010;**36**(9):518-20.
8. Clements M, Fuld J, Fritz Z. Documentation of resuscitation decision-making: a survey of practice in the United Kingdom. Resuscitation 2014;**85**(5):606-11.
9. Myint PK, Miles S, Halliday DA, et al. Experiences and views of specialist registrars in geriatric medicine on 'do not attempt resuscitation' decisions: a sea of uncertainty? QJM : monthly journal of the Association of Physicians 2006;**99**(10):691-700.
10. Smith GB, Poplett N, Williams D. Staff awareness of a 'Do Not Attempt Resuscitation' policy in a District General Hospital. Resuscitation 2005;**65**(2):159-63.
11. Saevareid TJ, Balandin S. Nurses' perceptions of attempting cardiopulmonary resuscitation on oldest old patients. Journal of advanced nursing 2011;**67**(8):1739-48.
12. Imhof L, Mahrer-Imhof R, Janisch C, et al. Do not attempt resuscitation: the importance of consensual decisions. Swiss medical weekly 2011;**141**:w13157.
13. Field RA, Soar J, Nolan JP, et al. Epidemiology and outcome of cardiac arrests reported in the lay-press: an observational study. Journal of the Royal Society of Medicine 2011;**104**(12):525-31.
14. Kim do Y, Lee KE, Nam EM, et al. Do-not-resuscitate orders for terminal patients with cancer in teaching hospitals of Korea. Journal of palliative medicine 2007;**10**(5):1153-8.
15. Field RA, Fritz Z, Baker A, et al. Systematic Review of Interventions to Improve Appropriate Use and Outcomes Associated with Do-Not-Attempt-Cardiopulmonary-Resuscitation Decisions. Resuscitation 2014 doi: 10.1016/j.resuscitation.2014.08.024. [Epub ahead of print]
16. Sosna DP, Christopher M, Pesto MM, et al. Implementation strategies for a do-not-resuscitate program in the prehospital setting. Annals of emergency medicine 1994;**23**(5):1042-6.
17. Butler MW, Saaidin N, Sheikh AA, et al. Dissatisfaction with Do Not Attempt Resuscitation Orders: A nationwide study of Irish consultant physician practices. Irish medical journal 2006;**99**(7):208-10.



For peer review only - http://bmjopen.bmj.com/site/about/guidelines.xhtml

## Figures and tables

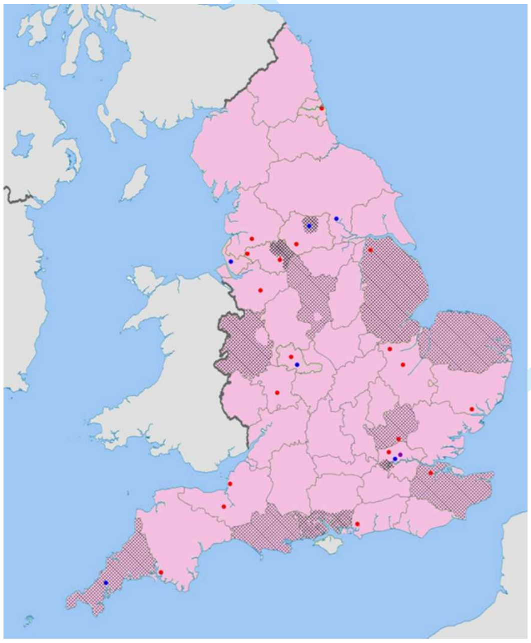

**Figure 1** Overview and geographic coverage of local Trust DNACPR policies included in the review

(•Teaching hospital; •District general hospital; • Specialist hospital; ▒ Community healthcare Trust)



For peer review only - http://bmjopen.bmj.com/site/about/guidelines.xhtml

**Table 1** Overview of variation in type of local DNACPR policies by Trust type

| **Policy type item** | **Acute Trusts (n=26), n (%)** | **Community Trusts (n=12), n (%)** | **AS Trusts (n=10), n (%)** | **Total, N=48, n (%)** |
|---|---|---|---|---|
| TYPE OF DOCUMENT | | | | |
| Policy | 22 (85%) | 12 (100%) | 7 (70%) | 41 (85%) |
| Procedure | 2 (8%) | 0 | 1 (10%) | 3 (6%) |
| Guideline | 2 (8%) | 0 | 2 (20%) | 4 (8%) |
| TERMINOLOGY | | | | |
| DNAR | 6 (23%) | 3 (25%) | 3 (30%) | 12 (25%) |
| DNACPR | 16 (62%) | 8 (67%) | 7 (70%) | 31 (65%) |
| Not for CPR | 3 (12%) | 0 | 0 | 3 (6%) |
| AND | 1 (4%) | 2 (17%)* | 0 | 3 (6%) |
| VISIBILITY | | | | |
| Stand alone | 21 (81%) | 9 (75%) | 3 (30%) | 33 (69%) |
| Integrated into resuscitation policy | 5 (19%) | 3 (25%) | 3 (30%) | 11 (23%) |
| Mentioned in other policies | 0 | 0 | 4 (40%) | 4 (8%) |
| COVERAGE | | | | |
| Local | 23 (88%) | 9 (75%) | 8 (80%) | 40 (83%) |
| Regional | 3 (12%) | 3 (25%) | 2 (20%) | 8 (17%) |

*Column does not add up to 12 because one Trusts consciously and consistently referred to DNACPR/AND throughout the policy
(Abbreviations: AS Ambulance service, DNAR do not attempt resuscitation, DNACPR do not attempt cardiopulmonary resuscitation, CPR cardiopulmonary resuscitation, AND allow natural death)



For peer review only - http://bmjopen.bmj.com/site/about/guidelines.xhtml

**Table 2** Overview of adoption of RC (UK) DNACPR forms and type of form by acute, community and AS Trusts

| **DNACPR form** | **Acute Trusts (n=26), n(%)** | **Community Trusts (n=12), n (%)** | **AS Trusts (n=10), n (%)** | **Total, N=48, n (%)** |
|---|---|---|---|---|
| RC (UK) | 2 (7.8%) | 3 (25%) | 0 | 5 (10.4%) |
| Modified | 4 (15.4%) | 3 (25%) | 1 (10%) | 8 (16.7%) |
| Bespoke | 16 (61.5%) | 4 (33.3%) | 4 (40%) | 24 (50%) |
| Form not sent | 4 (15.4%) | 2 (16.6%) | 5 (50%) | 11 (22.9%) |
| Type of form* | | | | |
| Paper | 24 (92.3%) | 10 (83.3%) | 5 (50%) | 39 (81.3%) |
| Electronic | 1 (3.8%) | 0 | 0 | 1 (2.1%) |
| Both | 1 (3.8%) | 2 (16.6%) | 0 | 3 (6.3%) |

*Insufficient information was available in policies from 5 Trusts for this question
(Abbreviations: RC (UK) Resuscitation Council (UK), DNACPR do not attempt cardiopulmonary resuscitation, AS ambulance service)

For peer review only

**Box 1** Example of clear pathway for handover of DNACPR decision from one acute trust

Review the appropriateness of the DNACPR decision before discharge

If on review the DNACPR is still considered appropriate carry decision over to the patient's care setting/home on discharge including the following considerations:

- Liaise with the patient's GP and identify an agreed, appropriate community review date (ideally within 24 hours), which should be documented on the DNACPR form
- Send the original DNACPR form (top white copy) with a review date in place with the patient
- Communicate and discuss sensitively with the patient
- Leave the yellow copy (second copy) in the front of the patient's medical hospital notes facilitating early consideration of resuscitation issues on any potential subsequent readmission

Communication with Ambulance Service and / or ongoing care setting must take place

Before Ambulance transfer complete appropriate section on the DNACPR form

When booking transport for DNACPR patient from acute Trust fax DNACPR form to ambulance control Contact Centre

Within community settings, hand DNACPR forms directly to the attending crew on arrival or fax to AS Contact Centre

The DNACPR status must also be recorded by the attending AS crew on the AS Patient Report Form

For peer review only

**Electronic supplementary material**

Table E1 Data extraction table for DNACPR policies received from Acute/ Ambulance services / and Community Care trusts

| Descriptor | Definition | Possible responses | |
|---|---|---|---|
| NHS type | What type of organization is it? | Teaching Hospital<br>District general<br>Community Trust<br>Ambulance | 1 |
| Decision maker | Is the organization a primary decision maker? | Policy refers to decision making<br>Policy refers to handling decisions | 2 |
| Policy type | Is the document a policy or guideline? | Policy / procedure / guideline | 3 |
| | What terminology is used? | DNAR / DNACPR / Not for CPR / AND | 4 |
| | What type of policy is it? | Stand-alone / integrated into resuscitation policy / mentioned in other policies | 5 |
| | Is it a local or unified policy? | Local / Unified | 6 |
| Date | When was the policy last reviewed (number of months from last review till month when policy was received)<br>Is policy within review cycle? | | 7 |



For peer review only - http://bmjopen.bmj.com/site/about/guidelines.xhtml

| | | | |
|---|---|---|---|
| | If NO is the policy under review? | Y / N / U<br><br>Y / N / U | 8<br><br>9 |
| Equality and diversity | Has an equality and diversity assessment been carried out? | Y / N / U | 10 |
| Law | Does the policy refer to relevant law / guidelines | Y / N / U<br><br>GMC<br>RC(UK) / BMA<br>Human rights act<br>Mental capacity act<br>JRCALC | 11 |
| Legal basis | Does the policy describe the situations in which DNACPR decision can be made | Futility<br>Benefit / Burden<br>Refusal | 12 |
| Assessment of capacity | Does the policy require staff to assess patient capacity? | Yes / No / Unclear | 13 |
| Communication / consultation | Does the policy tell staff when they should consult / inform patients / relatives / IMCA | Patient / LPA (Welfare Attorney, Deputy or Guardian) / Relatives / IMCA | 14 |
| Validity | Has the relationship between DNAR and other aspects of care been specified?<br><br>Is the decision valid indefinitely? | Y / N / U<br><br>Valid for one hospital admission / 'Indefinite' valid between admissions, after hospital discharge / Valid till specific time point<br><br>Not stated / Clinical | 15<br><br>16 |

For peer review only

| | | | |
|---|---|---|---|
| | What is the stated review period of the decision? | discretion / specific prompts (a) (re-)admission (b) at transfer or discharge (c) before surgery or anesthesia / regularly (in weeks/months) / change in patient status / patient change of mind | 17 |
| Multidisciplinary | Does the policy require clinician to consult a multi-disciplinary team | Must / Should / Does not mention | 18 |
| Portability | Is the decision valid outside the organisation?<br><br>If YES are clear systems described for portability / handover between healthcare providers? | Portable / Portable for AS only / Handover / No systems for portability or handover<br><br>Yes / No / Unclear | 19<br><br>20 |
| DNAR decision guidance | Does the policy contain practical flow diagram for best decision making from RC(UK) national guideline? | Yes / Modified / No | 21 |
| Diversity | Does the policy contain guidance relating to cultural differences? | Yes / No / Unclear | 22 |
| Signatories on form | Has the trust adopted the RCUK DNACPR form?<br><br>If NO who signs the DNAR forms?<br><br>Type of form | Yes / Modified / Bespoke<br><br>Consultant / GP / Doctor / Nurse / Patient / Relative / Other<br><br>Paper / Electronic / Both | 23 |
| Patient info leaflet | Does the policy refer to a patient information leaflet for DNACPR/resuscitation? | RC(UK) / local version / yes but not specified / No | 24 |
| Audit | Does the policy contain information on audit? | Yes | 25 |



For peer review only - http://bmjopen.bmj.com/site/about/guidelines.xhtml

| | | | |
|---|---|---|---|
| | | No | |
| Decision making | Has the consultant overall responsibility for decisions?<br><br>Which grade can make decisions? | Y / N / U<br><br>Consultant / GP / Nurse / Other doctor grade | 26 |

For peer review only

Table E2 Compliance with the requirement to refer to relevant laws / guidelines in local policies by acute, community and AS Trusts

| Item from national guidelines | Acute Trusts (n=26), n (%) | Community Trusts (n=12), n (%) | AS Trusts (n=10), n (%) | Total, N=48, n (%) |
|---|---|---|---|---|
| Has an equality and diversity assessment been carried out? | 17 (65.4%) | 10 (83.3%) | 9 (90%) | 36 (75%) |
| Does the policy describe all three situations (futility, burden, refusal) in which DNACPR decisions are appropriate? | 26 (100%) | 12 (100%) | 6 (60%) | 44 (91.7%) |
| Does the policy require staff to assess patient capacity? | 15 (57.7%) | 9 (75%) | 1 (10%) | 25 (52.1%) |
| Does the policy clarify the relationship between DNACPR and other aspects of care? | 25 (96.2%) | 11 (91.7%) | 9 (90%) | 45 (93.8%) |
| Does the policy contain the RCUK flow diagram to guide DNACPR decisions yes or modified? | 20 (41.7%) | 10 (83.3%) | 3 (30%) | 33 (68.8%) |
| Does the policy contain guidance relating to cultural differences? | 3 (11.5%) | 1 (8.3%) | 0 | 4 (8.3%) |



For peer review only - http://bmjopen.bmj.com/site/about/guidelines.xhtml

| | | | | |
|---|---|---|---|---|
| Does the policy refer to a patient information leaflet for DNACPR? yes [RC (UK)] | 12 [1] (46.2% [3.8%]) | 9 [0] (75%) | 1 (10%) | 22 [1] (45.8% [2.1%]) |
| Does the policy contain information on audit? | 26 (100%) | 12 (100) | 9 (90%) | 47 (97.9%) |

(Abbreviations: AS ambulance service, DNACPR do not attempt cardiopulmonary resuscitation, RC (UK) Resuscitation Council (UK))

For peer review only

Table E3 Compliance with the requirement to consult patients and relevant others by acute, community and AS Trusts

| Does the policy tell staff when to consult / inform patients or relevant others? | **Acute Trusts (n=26), n (%)** | **Community Trusts (n=12), n (%)** | **AS Trusts (n=10), n (%)** | **Total, N=48, n (%)** |
|---|---|---|---|---|
| Patients | 26 (100%) | 12 (100%) | 6 (60%) | 44 (91.7%) |
| LPA | 20 (41.7%) | 11 (91.7%) | 3 (30%) | 34 (70.8%) |
| Relatives | 26 (100%) | 11 (91.7%) | 5 (50%) | 42 (87.5%) |
| IMCA | 18 (69.2%) | 8 (66.7%) | 2 (20%) | 28 (58.3%) |

(Abbreviations: AS ambulance service, LPA lasting power of attorney, IMCA independent mental capacity advocate)

Table E4 Compliance with the requirement to consult a multidisciplinary team for decision a making by acute and community Trusts

| **Consultation of multidisciplinary team** | **Acute Trusts (n=26), n (%)** | **Community Trusts (n=12), n (%)** | **Total, N=38, n (%)** |
|---|---|---|---|
| Must | 1 (3.8%) | 2 (16.6%) | 3 (7.9%) |
| Should | 22 (84.6%) | 6 (50%) | 28 (73.7%) |
| Not mentioned | 3 (11.5%) | 4 (33.3%) | 7 (18.4%) |



For peer review only - http://bmjopen.bmj.com/site/about/guidelines.xhtml

**Policies on review of DNACPR decisions**

Policies on review ranged from regular review required with or without specification of a review date to regular review not recommended. The majority of policies included specific prompts that required clinicians to initiate review. These prompts included admission / readmission, transfer / discharge, surgery or anaesthesia and the change of the patient's condition or a change of heart.

A review period was given in 7/26 (27%) policies from acute trusts which ranged from 24 hours to 6 months. Six policies (23%) recommended regular review but did not specify a particular time period, while four (15%) policies did not recommend regular review. One stated that review was not required and eight (31%) did not mention regular review. Specific prompts for review were admission/readmission (n=17), transfer/discharge (n=16) and surgery/anaesthesia (n=16). Clinical discretion would prompt a review in 22/26 (85%) policies. And a change in the patient's condition or patient's expressed wishes as a reason for review was mentioned in 24/26 (92%) policies.

Similarly, regular review was mentioned in 5/12 policies from community trusts, which ranged from weekly (n=2) to 6 monthly (n=1) and not specified (n=2). One trust did not recommend regular review. The frequency of the review period was determined by clinical judgment and the change in patient status / patient change of mind in the majority of policies (8/12 and 11/12 respectively). Specific situations that prompted review were admission/readmission (7/12), transfer/discharge (9/12) and surgery/anaesthesia (4/12).

5/10 policies from ambulance services did not mention anything on review. 3/10 policies stated that if a review date is specified on the form, AS staff need to check whether the decision is within the review period. 5/10 policies stated that decisions without review date including decisions for terminal ill patients should be considered as indefinite. For other patients regular review is recommended in three of these five policies, while one policy does not recommend regular review. In 5/10 policies review is required at clinical discretion. Situations that should prompt a review were change in the patient's condition or change in expressed wishes (n=3), admission / readmission (n=1) and transfer / discharge (n=1). One policy recognised that review



For peer review only - http://bmjopen.bmj.com/site/about/guidelines.xhtml

dates on forms will vary across the region according to different local policies. And one policy included a form that stated that no decision should be regarded as indefinite and that the review period must be no longer than 3 months.

For peer review only



For peer review only - http://bmjopen.bmj.com/site/about/guidelines.xhtml
